# Desolvation-Driven 100-Fold Slow-down of Tunneling Relaxation Rate in Co(II)-Dy(III) Single-Molecule Magnets through a Single-Crystal-to-Single-Crystal Process

**DOI:** 10.1038/srep16621

**Published:** 2015-11-17

**Authors:** Jun-Liang Liu, Jie-Yi Wu, Guo-Zhang Huang, Yan-Cong Chen, Jian-Hua Jia, Liviu Ungur, Liviu F. Chibotaru, Xiao-Ming Chen, Ming-Liang Tong

**Affiliations:** 1 Key Laboratory of Bioinorganic and Synthetic Chemistry of Ministry of Education, School of Chemistry and Chemical Engineering, Sun Yat-Sen University, 510275 Guangzhou, Guangdong, P. R. China; 2 Theory of Nanomaterials Group and INPAC – Institute of Nanoscale Physics and Chemistry, Katholieke Universiteit Leuven, Celestijnenlaan 200F, 3001 Leuven, Belgium

## Abstract

Single-molecule magnets (SMMs) are regarded as a class of promising materials for spintronic and ultrahigh-density storage devices. Tuning the magnetic dynamics of single-molecule magnets is a crucial challenge for chemists. Lanthanide ions are not only highly magnetically anisotropic but also highly sensitive to the changes in the coordination environments. We developed a feasible approach to understand parts of the magneto-structure correlations and propose to regulate the relaxation behaviors via rational design. A series of Co(II)-Dy(III)-Co(II) complexes were obtained using *in situ* synthesis; in this system of complexes, the relaxation dynamics can be greatly improved, accompanied with desolvation, via single-crystal to single-crystal transformation. The effective energy barrier can be increased from 293 cm^−1^ (422 K) to 416 cm^−1^ (600 K), and the tunneling relaxation time can be grown from 8.5 × 10^−4^ s to 7.4 × 10^−2^ s. These remarkable improvements are due to the change in the coordination environments of Dy(III) and Co(II). *Ab initio* calculations were performed to better understand the magnetic dynamics.

The study of single-molecule magnets (SMMs) has been a popular research topic in the past two decades because SMMs represent promising materials for ultrahigh-density storage and quantum computing in spintronics^1^,^2^,^3^,^4^,^5^,^6^,^7^ and are exquisite prototypes for exploring the nature of magnetochemistry. The magnetic ground states, which are doubly degenerate, so-called “spin-up” and “spin-down” states, ensure that each of the single molecules can show either of the opposite magnetization signals. The spin can absorb and emit phonons to jump over the anisotropy energy barrier (*U*_A_) slowly between the magnetic bistable states or simply pass through via fast quantum tunneling of magnetization (QTM). To increase the blocking temperature (*T*_B_, defined as the temperature at which the relaxation time slows down to 100 s without external fields)^4^, it is critically important to both enhance the anisotropy energy barrier and cut off the tunneling pathway as far as possible^8^,^9^,^10^,^11^,^12^,^13^,^14^,^15^,^16^,^17^. Chemists are interested in determining the magneto-structure correlations and how to tune the intrinsic magnetism by utilizing chemical regulations.

Lanthanide ions have proved useful in the field of molecular magnetism, as they are the core of the highest performing mono- and poly-nuclear molecular magnets^18^,^19^,^20^. Particular examples of such lanthanide ions are Ishikawa’s [Tb(Pc)_2_]^0/− ^21; strong magnetization blocking displayed by the near-*C*_8_ symmetric [Er(COT)_2_]^−^ anion^22^,^23^, showing magnetic hysteresis at 10 K and magnetic remanence; the Zn-Dy-Zn compound^15^, where Dy(III) site has near-*D*_5h_ symmetry; and the lanthanide-radical Ln_2_N_2_^3−^ molecular magnets^24^,^25^.

The ground *J* manifold of the lanthanides could be regarded as the spin *S* in the case of classical spin magnets, such as Mn_12_Ac^1^. Large zero-field splitting of the latter is difficult to induce, i.e., the zero-field splitting parameter *D* is typically small, whereas the typical splitting of the ground *J* manifold of lanthanides in the absence of applied field is as large as several hundreds of wavenumbers (cm^−1^). Furthermore, the advantage of the crystal-field induced magnetic axiality in anisotropic Ln(III) ions compared with that induced by zero-field splitting in polynuclear complexes is that the environment surrounding a single metal (Ln) ion can be rationally designed, modified and adapted by using various terminal groups, solvents, or counterions. In this manner, the non-axial terms of the crystal-field Hamiltonian can be diminished, thus making them unimportant for the magnetic anisotropy in the low-lying states. We have recently found that quasi-*D*_5h_ environment of the Dy(III) ion in Zn-Dy-Zn^15^ and Fe-Dy-Fe^17^ compounds leads to strong magnetization blocking. Herein, we go beyond the previous analysis and attempt to understand the structural reasons for such strong magnetic blocking in a series of Co-Dy-Co complexes having similar quasi-*D*_5h_ symmetry of the Dy(III). Moreover, we demonstrate that the non-bonded co-crystallized water molecules influence directly the structural environment of the Dy(III) and Co(II) sites, having crucial consequences on increasing the magnetization-blocking barrier in this series as well as the magnetic relaxation time.

A series of [Co^II^_2_Dy^III^] single-molecule magnets, [Co_2_Dy(**L**^**Br**^)_2_(H_2_O)]NO_3_·3H_2_O (**1∙3H**_**2**_**O**, where **L**^**Br**^ = 2,2′,2″-(((nitrilotris(ethane-2,1-diyl))tris(azanediyl))tris(methylene))tris-(4-bromophenol)), [Co_2_Dy(**L**^**Br**^)_2_(H_2_O)]NO_3_·H_2_O (**1∙H**_**2**_**O**), and [Co_2_Dy(**L**^**Br**^)_2_(H_2_O)]NO_3_ (**1**), were isolated by *in situ* synthesis and single-crystal to single-crystal transformation. Accompanied with the desolvation, the effective anisotropy energy barrier (*U*_eff_) increases from 293 cm^−1^ (422 K) to 416 cm^−1^ (600 K), which is the highest value reported for all *d*-*f* molecules^17^, and the tunneling relaxation time (*τ*_QTM_) dramatically increases by two orders of magnitude, from 8.5 × 10^−4^ s to 7.4 × 10^−2^ s. In this paper, we discuss the structural changes of the coordination environment of the Dy(III) and Co(II) sites, which significantly affect the anisotropy energy barriers and the QTM process.

All molecular structures of this [Co^II^_2_Dy^III^] series contain a 7-coordinate (compressed pentagonal bipyramid, *D*_5h_) Dy(III) ion at the center and two asymmetric 6-coordinate Co(II) at the two sides (Fig. 1). Upon removing the outer water molecules, the bond between Dy(III) and the terminal water molecule becomes significantly longer, from 2.419(4) to 2.508(19) Å, as presented in Table 1. The five equatorial atoms are more coplanar, as indicated by the decrease of the standard deviation of the distance to its least-squares plane, from 0.226 to 0.076 Å. Thus, the geometry of Dy(III) for **1** deviates less than that of **1∙3H**_**2**_**O** (see the Continuous Symmetry Measures (CSM) values calculating by program SHAPE)^26^. Furthermore, the geometries of the two asymmetric Co(II) are transformed from distorted octahedron (*O*_h_) to the intermediate between octahedron and trigonal prism (*D*_3h_) according to the CSM values, and both of Dy-Co1 and Dy-Co2 distances increase slightly by 0.05–0.08 Å.

Temperature-dependent magnetic susceptibility measurements were performed on the samples (Fig. 2), whose *χ*_M_*T* value at 300 K were 19.7 and 19.2 cm^3^ mol^−1^ K for **1∙3H**_**2**_**O** and **1**, respectively, which are somewhat higher than the expected values (17.9 cm^3^ mol^−1^ K, Dy(III): ^6^*H*_15/2_, *J* = ^15^/_2_, *g* = ^4^/_3_; Co(II): *S* = ^3^/_2_, *g* = 2)^27^. On cooling, all of the products gradually decrease to the minima (**1∙3H**_**2**_**O**: 16.8 cm^3^ mol^−1^ K at 4.1 K; **1**: 17.1 cm^3^ mol^−1^ K at 7.0 K) and then very slightly increase (**1∙3H**_**2**_**O**: 16.9 cm^3^ mol^−1^ K at 1.8 K; **1**: 17.5 cm^3^ mol^−1^ K at 1.8 K), suggesting the coexistence of the crystal-field splitting and/or spin-orbit coupling for Dy(III) and Co(II) as well as the weak ferromagnetic interactions between them.

Measurements of the ac susceptibilities were performed to probe the magnetic dynamics of [Co^II^_2_Dy^III^] compounds (Fig. 3). Temperature- and frequency-dependent ac signals of these compounds indicate slow magnetic relaxation under zero field. These signals were fitted using a generalized Debye model (Figure S5)^28^, revealing narrow distributions of the relaxation times (*α* parameters: 0.05–0.10 for **1∙3H**_**2**_**O** and 0.00–0.13 for **1**).

In general, the relaxation rate (*τ*^−1^) is a combination of the Orbach process (*τ*_Orbach_^−1^ ~ exp(−*U*_eff_/*k*_B_*T*)), the direct and Raman process (*τ*_Orbach_^−1^ ~ *T*^n^; *n* = 1 for direct process, 4 ≤ *n* ≤ 9 for typical Raman process), and the QTM process (*τ*_QTM_^−1^)^13^,^29^,^30^,^31^.


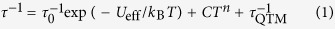


The best fits for all compounds obtained using this model are displayed in Fig. 4 and Table 2. These three different mechanisms of magnetic relaxation are dominant at high-, intermediate-, and low-temperature regimes, respectively, giving rise to three trends: 1. Enhancing anisotropy barriers, 293 cm^−1^ (422 K) for **1∙3H**_**2**_**O** < 416 cm^−1^ (600 K) for **1**; 2. Weakening direct process, *n* = 1 for **1∙3H**_**2**_**O** vs. *n* = 3.2 for **1**; 3. Increasing tunneling relaxation time, 8.5  × 10^−4^ s for **1∙3H**_**2**_**O** < 7.4 × 10^−2^ s for **1**. These noteworthy changes of magnetic dynamics are discussed below.

For the partially desolvated **1∙H**_**2**_**O**, two sets of temperature-dependent and frequency-dependent ac signals are observed (Figures S4–S6), of which the fast-relaxation (FR) regime and slow-relaxation (SR) regime are similar to those of **1∙3H**_**2**_**O** and **1**, respectively (Figure S7 and Table 2). The differences may be caused by tiny structural changes (inherent nature), doping of each other (magnetic dipolar interactions) and measurement/fitting errors (especially the high-temperature regime). As single-crystal X-ray diffraction can only provide an average structure, determining the difference between **1∙H**_**2**_**O** and the other two structures is not guaranteed. However, the discrete relaxation dynamics in **1∙H**_**2**_**O** confirms that the outer environment of the molecules indeed results in distinct magnetic behaviors, which we discuss based on the corresponding solvated (**1∙3H**_**2**_**O**) and desolvated (**1**) analogs.

*Ab initio* calculations of the CASSCF/RASSI-SO/SINGLE_ANISO type using the MOLCAS program package^18^,^32^ were performed to determine the local electronic and magnetic properties on individual metal sites. We refer to the ESI for the computational details and the complete set of results. Here, we discuss the main findings.

As expected, Dy(III) sites in **1** and **1·3H**_**2**_**O** were found to have strong uniaxial magnetic anisotropy in their ground doublet states (Fig. 1). Table 3 presents the *ab initio* calculated low-lying energy spectrum on metal sites in the investigated compounds, and Table 4 provides information related to magnetic anisotropy in the ground and first-excited Kramers doublet (KD) states. The obtained magnetic axiality is comparable with the magnetic anisotropy of the Dy(III) in the Zn(II)-Dy(III)-Zn(II)^15^ and Fe(II)-Dy(III)-Fe(II)^17^ compounds, where the main anisotropy axes on Dy(III) had similar orientations. We can see from Table 4 that the transversal *g*-factors for the first excited KD are much smaller in **1** compared to **1·3H**_**2**_**O**, implying a higher axiality of the excited states in the former compound. Therefore, while the activation barrier corresponds to the first excited KD in **1·3H**_**2**_**O**, it corresponds to a higher KD in **1**, which explains the observed large discrepancy in the relaxation barriers of two compounds (Table 2).

To understand the obtained strong magnetic axiality of the Dy(III) in this coordination environment, we analyzed the main structural features underlying the strongest axial effect on this metal site. Table 1 presents the main structural differences between the investigated compounds.

The main anisotropy axis is oriented along the two shortest bonds of the Dy(III) – along the axis connecting oxygen atoms O1 and O4. The short chemical bond means the strong covalent effect arising from these two oxygen atoms. Moreover, oxygen atoms O1 and O4 hold the largest negative electronic charge. The calculated LoProp charges^33^ are listed in Tables S7 and S8. This result demonstrates that the total axial crystal-field effect on Dy(III), comprising both covalent effect (short chemical bond) and electrostatic (largest negative charge) from the oxygen atoms O1 and O4, is dominant, defining the orientation of the magnetic axis in these compounds. Indeed, because the axial crystal-field effect of any other ligand atoms cannot overcome the effect of the O1 and O4 atoms (if that was the case, the anisotropy axis would be rotated from the obtained direction), the main effect of all remaining atoms is to induce non-axial crystal-field contributions.

Using the recently developed tool in SINGLE_ANISO, we extracted all of the parameters 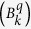 of the crystal field from the *ab initio* calculations.


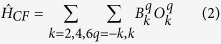


Table S6 contains the full set of *ab initio* extracted crystal-field parameters. Analyzing the splitting induced by individual parameters of each rank, the second-rank terms induce the largest crystal-field splitting, although the fourth-order ones are also important. In particular, the 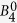 axial parameter imposes the high axiality of the ground doublet state and the large energy separation between the ground and the first-excited doublet states.

The first coordination environment of Co(II) in **1** and **1·3H**_**2**_**O** is far from any point group symmetry. However, Table 3 illustrates the tendency that the zero-field splitting of the ground spin state *S* = ^3^/_2_ of both Co(II) sites in **1** is significantly smaller than the zero-field splittings of these sites in **1·3H**_**2**_**O**. Because the size of the zero-field splitting is directly proportional to the effect of spin-orbit coupling, we may assume that the unquenched orbital momentum on the Co(II) sites in **1** is smaller than that in **1·3H**_**2**_**O**. Indeed, these assumptions are found to be qualitatively correct from Table 4.

The origin of stronger quenching of angular momentum in **1** can be understood by the larger “*D*_3h_” character according to calculated CSM values, which would ideally stabilize a non-degenerate ^4^*A*_1_ electronic ground state, implying that the spin-orbit coupling effect arises only in the second order of the perturbation theory. By contrast, both Co(II) sites in **1·3H**_**2**_**O** exhibit a larger “*O*_h_” character according to calculated CSM values, which means that the ground state is ideally triply degenerate ^4^*T*_1g_, involving the spin-orbit coupling already in the first order of perturbation theory.

The obtained *ab initio* results for individual metal sites were further used for the computation of the exchange spectrum and the magnetic properties of the trinuclear complexes using the POLY_ANISO program^18^,^34^. The exchange interactions of Dy(III)-Co(II) and Co(II)-Co(II) are considered within the Lines model^35^ (see ESI for more details), whereas the contribution of the intramolecular dipole-dipole magnetic coupling is precisely accounted for because all of the necessary data are available from the *ab initio* calculations. The best fitting parameters of the Lines model for the investigated compounds are presented in Table 5. All of the macroscopic magnetic properties were computed based on the resulting exchange spectrum of the complex. The estimation of the exchange couplings via broken-symmetry density functional theory (BS-DFT) calculations used within the ORCA 3.0.0 program^36^ (see ESI for more details) on isostructural CoGdCo (using the experimental structures of **1** and **1·3H**_**2**_**O**) provided slightly overestimated values.

The total magnetic interaction (exchange + dipolar) between the lowest Kramers doublets on the sites can be cast in a good approximation by the non-collinear Ising Hamiltonian:


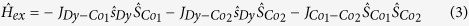


where 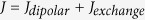 is the parameter of total magnetic interaction between metal sites, 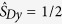 is the pseudospin of the ground doublet state of the Dy(III), and 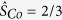 is the spin corresponding to the Co(II) sites.

The low-lying exchange doublets arising from the magnetic interactions of the ground manifold of two Co(II) sites (*S* = ^3^/_2_, split by ZFS) and the ground doublet 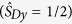 on the Dy(III) ((2 × ^3^/_2_ + 1)^2^ × (^1^/_2_ × 2 + 1) = 32, 16 doublets) are quite axial (*g*_X_,_Y_ < 10^−2^, see the ESI), implying a partially suppressed QTM in the tunneling regime, which is established at very low temperature, when only the ground exchange doublet (with zero energy in Table 5) is effectively populated. This regime was not achieved in the present work because of measuring temperature exceeding 2 K.

Magnetic relaxation in the high temperature regime, *i.e.*, at temperatures much higher than the exchange splitting, occurs *via* intraionic Orbach mechanism involving local excited Kramers doublets of the Dy(III) only. Due to high activation energy for this relaxation, its rate decreases quickly with lowering the temperature, so that at *T* < 20 K another relaxation mechanism becomes dominant, as can be seen in Fig. 4. The weak temperature dependence of ln(*τ*) in this domain suggests a relaxation of QTM type, which involves again intraionic transitions on Dy(III) sites only. The reason why the relaxation remains of intraionic character is the still relatively high temperature, exceeding much the exchange splitting in both compounds (Table 5). Given that Dy(III) is a Kramers ion, it requires the presence of a transversal magnetic field (directed in one of the two perpendicular directions to its main magnetic axis, see Fig. 1) to induce tunneling splitting and QTM relaxation. This is provided by the neighbor Dy(III) and Co(II) ions, which are sources of oscillating magnetic field^37^. It is natural to assume that the cobalt ions within the same complex will produce a stronger splitting of the ground KD of Dy(III) due to its small distance to the latter, giving rise also to the exchange interaction (see Table 5). However, the close resemblance of the relaxation curves for **1** in Fig. 4 and for the isostructural ZnDyZn complex^15^ (cf. Fig. 4 in the latter reference) points at a more significant role played by the neighbor Dy(III) ions in this relaxation process. This could be due to the fact that the magnetic moment on Co(II) flips much faster than on Dy(III) thus diminishing the effect from the cobalt ions.

The main challenge is to understand the large difference in the relaxation times of **1** and **1·3H**_**2**_**O** in the QTM regime (Fig. 4). The *ab initio* results in Tables 4 and 5 show close anisotropic properties of the ground Kramers doublets of Dy(III) in the two compounds and similar interaction constants with neighbor ions, which cannot explain the difference of two orders of magnitude of the respective *τ*_QTM_ (Table 2). Moreover, the spectra of excited Kramers doublets in Table 3 look also quite similarly for both complexes, implying close strengths of spin-phonon coupling. The only visible difference between the two compounds that could affect significantly their relaxation properties is the presence of additional three water molecules in **1·3H**_**2**_**O.** These enhance the vibrational coupling between different CoDyCo units in the crystal thus providing a better relaxation of magnetization. On the contrary, the absence of the lattice water molecules in **1** causes a phonon bottleneck effect thus suppressing the relaxation.

We now provide some paths along which further enhancement of magnetic behavior can occur. First, the magnetic axiality in the low-lying doublet states of the Dy(III) can be increased together with the axial components of the crystal field by shortening the two axial Dy-O1 and Dy-O4 chemical bonds and by increasing the O1-Dy-O4 angle up to 180°. Furthermore, the non-axial crystal field components can be reduced if weaker equatorial ligands are used, with nearly neutral bonding atoms. Minimization of the equatorial field can be achieved by eliminating all equatorial ligands, thus achieving a *D*_∞h_ local symmetry, albeit this is not that easy to achieve.

Another path to achieve performant molecular magnets is to couple strongly one Ising ion, such as Dy(III) in the present system, with two purely isotropic paramagnetic sites, such as a radical, Mn(II), or Fe(III). Such trinuclear compound would behave as a performant molecular magnet because of a multistep character of the blocking barrier. Tunneling will be suppressed at temperatures below the blocking barrier due to large number of intermediate steps required to connect states with opposite magnetization, whereas above this exchange barrier, the large spins on neighboring metal sites will be again a destructive factor for the magnetization blocking.

Finally, a particularly important conclusion emerging from the present work is that special care should be taken in order to diminish the contacts between the complexes in the crystal. Such contacts are promoted by species like water and probably other solvent molecules capable to bind the magnetic units via hydrogen bonds, thus contributing to more efficient heat transfer in the crystal and a faster magnetic relaxation.

In summary, we reported a series of linear Co(II)-Dy(III)-Co(II) single-molecule magnets with a Dy(III) in pentagonal-bipyramid geometry. The loss of the uncoordinated water molecules alters the effective energy barrier (>100 cm^−1^ increase) and the tunneling relaxation time (>100-fold growth) significantly, giving rise to a desolvated molecule with the highest known effective energy barrier for *d*-*f* molecules. These pronounced change of relaxation dynamics can contribute to the change in the coordination environments of Dy(III) and Co(II). These factors were understood qualitatively/quantitatively with the aid of *ab initio* calculations.

## Methods

All procedures were conducted under an inert N_2_ atmosphere by using Schlenk techniques.

### Synthesis of [Co_2_Dy(L^Br^)_2_(H_2_O)]NO_3_∙3H_2_O (1∙3H_2_O)

5-bromo-salicylaldehyde (0.6 mmol, 120 mg), tris(2-aminoethy1)amine (0.2 mmol, 30 mg) and 30 mL of methanol were mixed in a Schlenk flask. After adding NaBH_4_ (~30 mg) into the resulting yellow solution, the mixture was stirred for ~10 min until the solution turned to colorless, and then, dinitrogen was bubbled for additional 20 min. The solution was added to the mixture of Dy(NO_3_)_3_∙6H_2_O (0.1 mmol, 46 mg) and Co(NO_3_)_2_∙6H_2_O (58 mg), and then, triethylamine (~75 μL) was added dropwise into the solution. After stirring for 1 min and then slowly flowing dinitrogen for 3 h, light-pink crystals were grown in the remaining solution (~25 mL). The crystals were placed into a high-moisture atmosphere (up to 100% humidity at room temperature) for 2–3 h; ~80 mg light-pink crystals of **1∙3H**_**2**_**O** were obtained after filtration (~43% yield based on Dy). Elemental analysis (calcd, found): C (35.81, 36.08), H (3.78, 3.79), N (6.96, 6.92). IR(KBr, cm^−1^): 3626s, 3397m, 3240s, 3198s, 2907s, 2862s, 1585s, 1470vs, 1410s, 1383s, 1305vs, 1268vs, 1182m, 1158m, 1120m, 1091m, 1076m, 1032m, 999m, 959m, 884m, 826s, 784s, 744m, 636s, 534m, 472m, 454m, 404m.

### Synthesis of [Co_2_Dy(L^Br^)_2_(H_2_O)]NO_3_∙H_2_O (1∙H_2_O) and [Co_2_Dy(L^Br^)_2_(H_2_O)]NO_3_ (1)

**1∙H**_**2**_**O** was obtained after the crystals of **1∙3H**_**2**_**O** were in a slow rate of dinitrogen flow for 0.5 h; after an additional 2.5 h, **1∙H**_**2**_**O** was further transformed into **1** completely. Elemental analysis (calcd, found) for **1∙H**_**2**_**O**: C (36.54, 36.55), H (3.63, 3.93), N (7.10, 7.26). IR(KBr, cm^−1^): 3624s, 3417m, 3242s, 3201s, 2907s, 2864s, 1585s, 1470vs, 1410s, 1384s, 1305vs, 1270vs, 1182m, 1159m, 1121m, 1091m, 1077m, 1033m, 1000m, 959m, 884m, 826s, 786s, 742m, 638s, 535m, 472m, 458m, 404m. Elemental analysis (calcd, found) for **1**: C (36.91, 36.79), H (3.56, 3.62), N (7.18, 7.12). IR(KBr, cm^−1^): 3625s, 3394m, 3242s, 3201s, 2908s, 2863s, 1585s, 1470vs, 1410s, 1383s, 1305vs, 1268vs, 1182m, 1158m, 1120m, 1091m, 1076m, 1032m, 999m, 959m, 884m, 826s, 784s, 744m, 636s, 534m, 472m, 454m, 404m.

### X-ray Crystallographic Study

Diffraction intensities were collected using an Oxford Diffraction Gemini R CCD diffractometer with Cu*K*_α_ radiation (*λ* = 1.54178 Å) for **1∙3H**_**2**_**O**, **1∙H**_**2**_**O** and **1** at 150 K. The structures were solved by direct methods, and then refined using the SHELXTL program^38^. CCDC 1058028 (**1∙3H**_**2**_**O**) 1058029 (**1∙H**_**2**_**O**) and 1058030 (**1**) contain the supplementary crystallographic data for this paper. These data can be obtained free of charge from The Cambridge Crystallographic Data Centre via www.ccdc.cam.ac.uk/data_request/cif.

### Magnetic measurements

Magnetic susceptibility measurements were performed using a Quantum Design MPMS-XL7 SQUID. Polycrystalline samples were embedded in Vaseline to prevent torquing. Data were corrected for the diamagnetic contribution calculated from the Pascal constants.

## Additional Information

**How to cite this article**: Liu, J.-L. *et al.* Desolvation-Driven 100-Fold Slow-down of Tunneling Relaxation Rate in Co(II)-Dy(III) Single-Molecule Magnets through a Single-Crystal-to-Single-Crystal Process. *Sci. Rep.* **5**, 16621; doi: 10.1038/srep16621 (2015).

## Supplementary Material

Supplementary Information

## Figures and Tables

**Figure 1:**
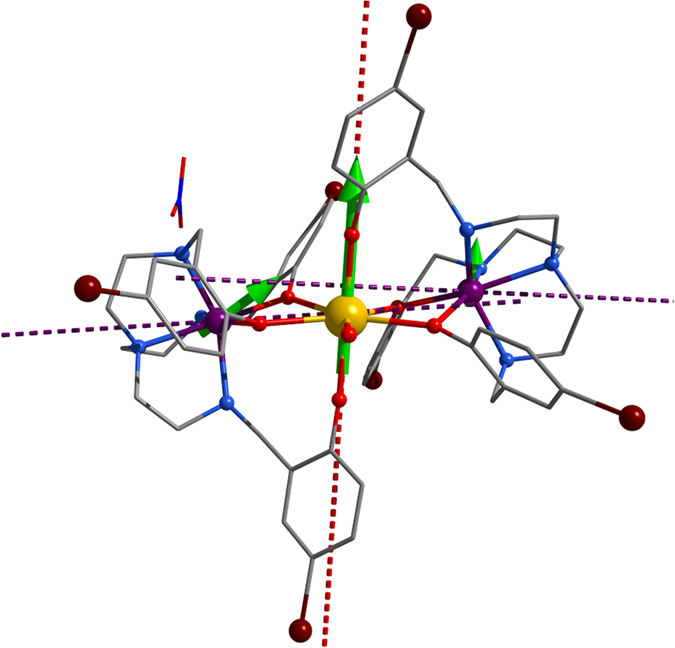
Molecular structure of 1. Color Codes: Dy, gold; Co, violet; Br, brown; O, red; N, blue; C, silver. Dashed lines represent the main anisotropy axes *g*_Z_ on the individual metal sites. Green arrows show the orientation of magnetic moments in the ground exchange state. The deviations of the local moments on Co(II) sites from their local *z* direction (dashed) is due to relatively strong ferromagnetic Ising-type exchange interaction with the Dy(III) site. The magnetic moment on the Co(II) site is particularly small in the ground exchange state. The reduction of the magnetic moment arises from the Ising-type exchange interaction with the Dy(III), which conserves the moment in the *z* direction of the latter.

**Figure 2:**
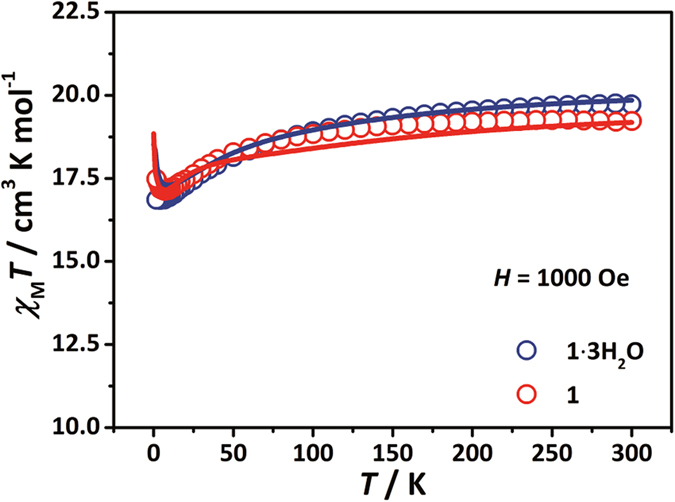
Temperature dependencies of χ_M_*T* products for 1∙3H_2_O and 1. The temperature range is 1.8 K ≤ *T* ≤ 300 K, and the applied field of 1,000 Oe.

**Figure 3:**
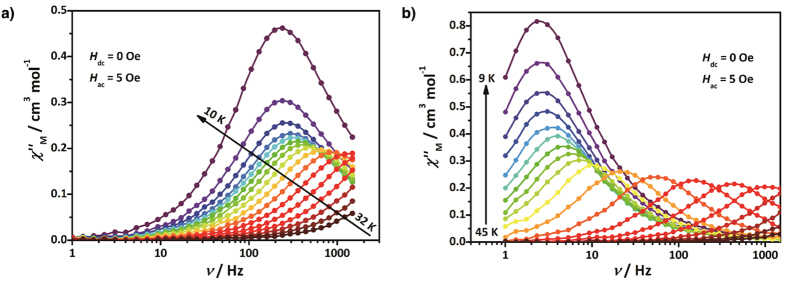
Out-of-phase ac magnetic susceptibilities (χ_M_”) for [Co^II^_2_Dy^III^]. (**a**) Out-of-phase susceptibility for **1∙3H**_**2**_**O** in *H*_dc_ = 0 Oe and *H*_ac_ = 5 Oe. (**b**) Out-of-phase susceptibility for **1** in *H*_dc_ = 0 Oe and *H*_ac_ = 5 Oe. All solid lines are visual guides.

**Figure 4:**
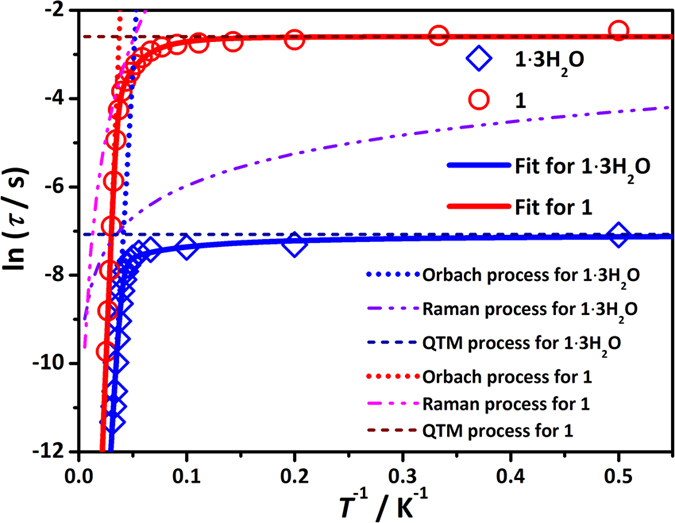
Magnetic relaxation dynamics for [Co^II^_2_Dy^III^]. Relaxation behaviors for **1∙3H**_**2**_**O** and **1**. The solid lines represent the best fits for the relevant compounds.

**Table 1:** Selected parameters of the molecular structures.

	1·3H_2_O	1
CSM Dy (*D*_5h_)	1.169	0.492
CSM Co1 (*O*_h_, *D*_3h_)	3.325, 8.732	4.451, 8.308
CSM Co2 (*O*_h_, *D*_3h_)	2.366, 9.575	6.611, 6.778
Dy-O1_AXIAL_ bond length/Å	2.175(5)	2.175(13)
Dy-O4_AXIAL_ bond length/Å	2.198(5)	2.171(15)
Average bond length of the five equatorial Dy-O/Å	2.387	2.379
Dy-O_water_ bond length/Å	2.419(4)	2.508(19)
Angle O1_AXIAL_-Dy- O4_AXIAL_	169.7°	169.8°
Standard deviation for the distance between the five equatorial oxygens and their least-square plane (σ/Å)	0.2257	0.0756
Dy-Co1 distance/Å	3.5426(12)	3.592(4)
Dy-Co2 distance/Å	3.5704(12)	3.646(4)

**Table 2:** Summary of the ac magnetic data.

	1·3H_2_O	1·H_2_O	1
τ_0_/s	2.4 × 10^−11^	3.5 × 10^−10^ (FR); 1.8 × 10^−10^ (SR)	1.4 × 10^−11^
*U*_eff_ / cm^−1^, K	293, 422	321, 462 (FR); 363, 522 (SR)	416, 600
τ_QTM_/s	8.5 × 10^−4^	1.4 × 10^−3^ (FR); 6.5 × 10^−2^ (SR)	7.4 × 10^−2^
*n*	1.0	2.8 (FR); 2.9 (SR)	3.2
*C*/s^−1^ K^−n^	36	0.12 (FR); 2.3 × 10^−3^ (SR)	1.1 × 10^−3^

**Table 3:** Calculated low-lying energy spectra (cm^−1^) of the individual Dy(III) and Co(II) sites in 1 and 1·3H_2_O.

KD	1			1·3H_2_O		
	Co1	Dy	Co2	Co1	Dy	Co2
1	0.0	0.0	0.0	0.0	0.0	0.0
2	62.2	292.5	44.0	93.8	294.5	128.5
3	1740.1	327.8	2315.0	1156.1	313.2	898.1
4	1884.6	404.7	2427.5	1354.0	404.2	1113.1
5	2881.8	485.5	3960.6	2097.6	466.5	1995.9
6	2976.8	543.4	4045.6	2206.4	494.9	2087.1
7		640.6			572.0	
8		684.9			599.3	

**Table 4:**
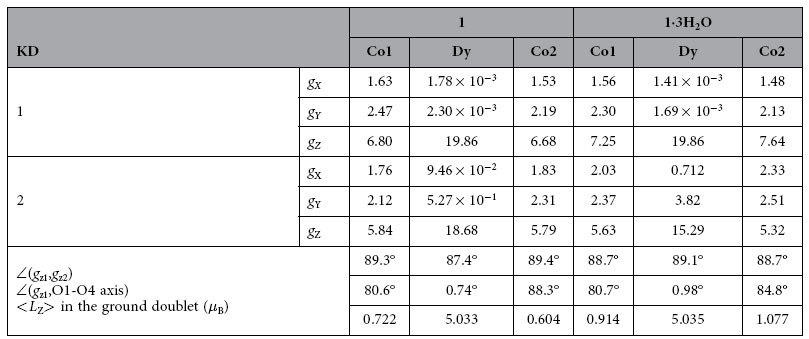
Calculated *g*-tensors of the ground and first-excited Kramer doublets and the angles between their main anisotropy axes of the individual Dy(III) and Co(II) sites in 1 and 1·3H_2_O.

**Table 5:** Exchange coupling constants of the investigated compounds extracted from the fitting of the measured magnetic data and obtained from broken-symmetry DFT calculations (in cm^−1^).

Exchange parameters	1		1∙3H_2_O	
	Lines (in cm^−1^)	BS-DFT (in cm^−1^)	Lines (in cm^−1^)	BS-DFT (in cm^−1^)
Dy-Co1	0.980	0.159	0.770	0.386
Dy-Co2	0.280	0.518	0.930	0.697
Co1-Co2	−0.380	−0.234	−0.560	−0.119
Low-lying energy spectrum	0.000	0.000	0.000	0.000
	0.345	0.178	1.053	0.848
	2.254	1.113	2.741	1.307
	3.002	1.625	4.088	2.242
	44.072	43.966	94.259	94.022
	44.676	44.358	95.136	94.650
	46.485	45.224	96.462	95.289
	47.279	45.634	97.775	96.019
	62.440	62.345	128.206	128.161
	62.600	62.439	129.855	129.083
	64.554	63.450	131.213	130.317
	65.040	63.662	133.202	131.286
	…	…	…	…
	***g* tensor in the ground exchange doublets**			
	1.1 × 10^−3^	1.3 × 10^−3^	3.0 × 10^−4^	3.0 × 10^−4^
	3.6 × 10^−3^	1.7 × 10^−3^	5.0 × 10^−4^	2.4 × 10^−3^
	24.55	21.26	24.34	24.76
