# Supplementary material for: Desolvation-Driven 100-Fold Slow-down of Tunneling Relaxation Rate in Co(II)-Dy(III) Single-Molecule Magnets through a Single-Crystal-to-Single-Crystal Process
Source: Sci Rep. 2015 Nov 17;5:16621. doi: 10.1038/srep16621 (PMC4647839; doi:10.1038/srep16621)
Supplement: Supplementary Information [file srep16621-s1.doc]

Supplementary Information

**Desolvation-Driven 100-Fold Slow-down of Tunneling Relaxation Rate in Co(II)-Dy(III) Single-Molecule Magnets through a Single-Crystal-to-Single-Crystal Process**

Jun-Liang Liu^1,†^, Jie-Yi Wu^1,†^, Guo-Zhang Huang^1^, Yan-Cong Chen^1^, Jian-Hua Jia^1^, Liviu Ungur^2^*, Liviu F. Chibotaru^2^, Xiao-Ming Chen^1^, and Ming-Liang Tong^1^*

† Key Laboratory of Bioinorganic and Synthetic Chemistry of Ministry of Education, School of Chemistry and Chemical Engineering, Sun Yat-Sen University, 510275 Guangzhou, Guangdong, P. R. China

# Theory of Nanomaterials Group and INPAC – Institute of Nanoscale Physics and Chemistry, Katholieke Universiteit Leuven, Celestijnenlaan 200F, 3001 Leuven, Belgium

‡ These authors contributed equally to this work.

*e-mails: tongml@mail.sysu.edu.cn; Liviu.Ungur@chem.kuleuven.be

Contents

[1. Crystal Data and Structure 2](#__RefHeading___Toc414545880)

[2. Magnetic Measurements 3](#__RefHeading___Toc414545881)

[3. Ab initio calculations 5](#__RefHeading___Toc414545882)

[Computational details 5](#__RefHeading___Toc414545883)

[Ab initio calculated parameters of the crystal-field for Dy^3+^ sites 9](#__RefHeading___Toc414545884)

[Calculated atomic charges 10](#__RefHeading___Toc414545885)

[Modelling of the exchange interactions 15](#__RefHeading___Toc414545886)

# 1. Crystal Data and Structure

**Table S1. Crystal Data and Structure Refinement**.

|  | **1·3H_2_O** | **1·H_2_O** | **1** |
| --- | --- | --- | --- |
| **Chemical Formula** | C_54_H_68_Br_6_Co_2_DyN_9_O_13_ | C_54_H_64_Br_6_Co_2_DyN_9_O_11_ | C_54_H_62_Br_6_Co_2_DyN_9_O_10_ |
| **Formula Mass / g mol^-1^** | 1810.99 | 1774.96 | 1756.95 |
| **Temperature / K** | 150 | 223 | 173 |
| ***a* / Å** | 21.7486(3) | 20.4911(13) | 20.7951(7) |
| ***b* / Å** | 15.9104(2) | 16.2125(7) | 16.1471(4) |
| ***c* / Å** | 36.9264(3) | 38.245(2) | 37.4504(13) |
| **Unit Cell Volume / Å^3^** | 12777.6(3) | 12705.4(13) | 12575.1(7) |
| ***Z*** | 8 | | |
| **Crystal System** | Orthorhombic | | |
| **Space Group** | *Pbca* | | |
| ***ρ*_calcd_ / g cm^-3^** | 1.883 | 1.856 | 1.856 |
| **μ(Cu*K*_α_) / mm^-1^** | 15.133 | 15.181 | 15.319 |
| ***R*_1_*^a^*(*I* > 2*σ*(*I*))** | 0.0490 | 0.1074 | 0.1378 |
| ***wR*_2_*^b^* (all data)** | 0.1229 | 0.3132 | 0.3152 |
| **Goodness of fit on *F*^2^** | 0.987 | 1.043 | 1.123 |

*^a^R*_1_ =Σ||*F*_o_| - |*F*_c_||/Σ|*F*_o_|, *^b^wR*_2_ = [Σ*w*(*F*_o_^2^-*F*_c_^2^)2/Σ*w*(*F*_o_^2^)^2^]^1/2^


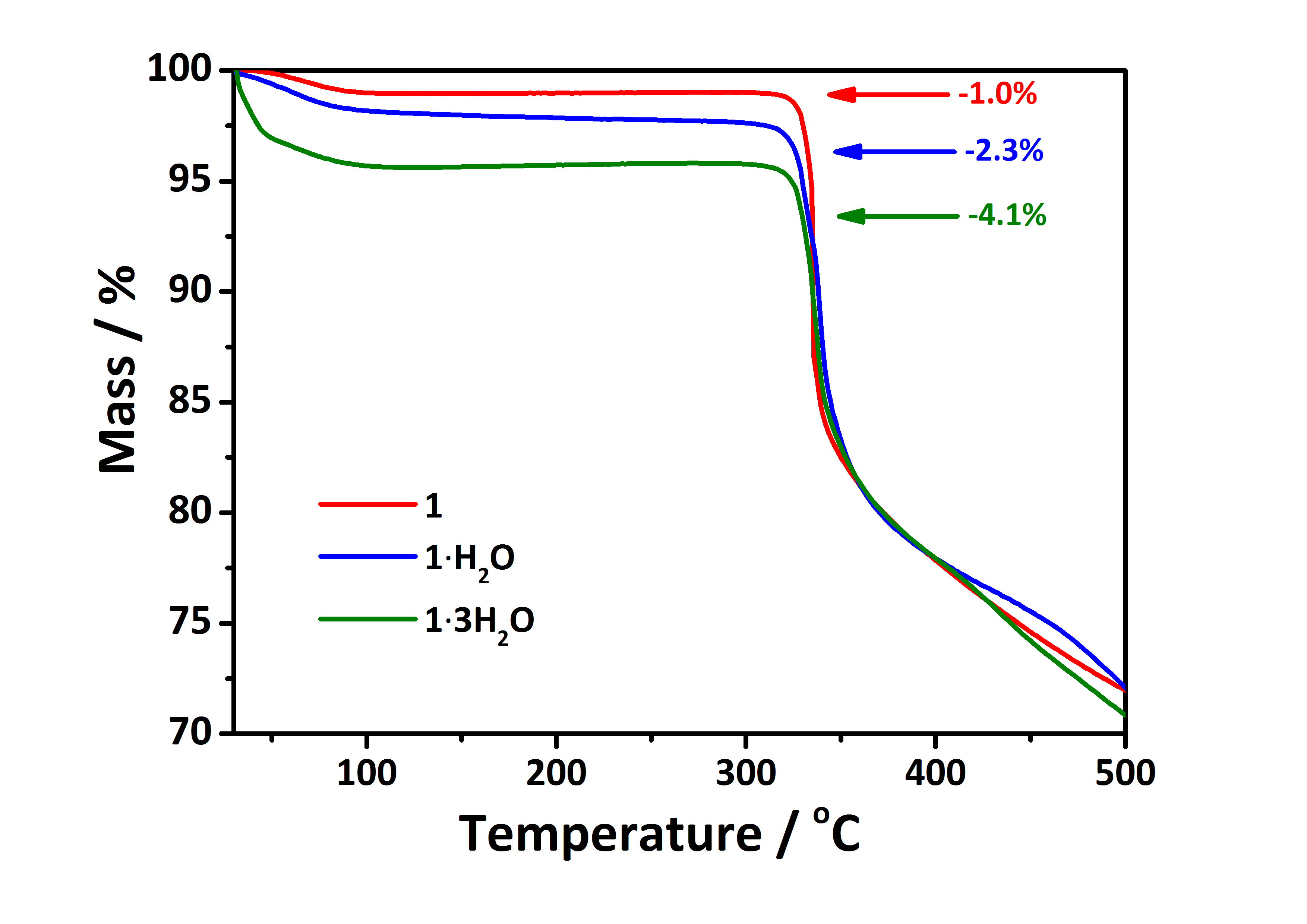


**Figure S1 | Thermogravimetric analysis for** **1, 1∙H_2_O and 1∙3H_2_O.** The loss weight of 1.0%, 2.3% and 4.1 % below 300 ^o^C match the expected loss weight of 1.0 %, 2.0 % and 4.0 %, including 1 coordinated and uncoordinated water molecules (0, 1 and 3), for **1**, **1∙H_2_O** and **1∙3H_2_O**, respectively.

# 2. Magnetic Measurements

**
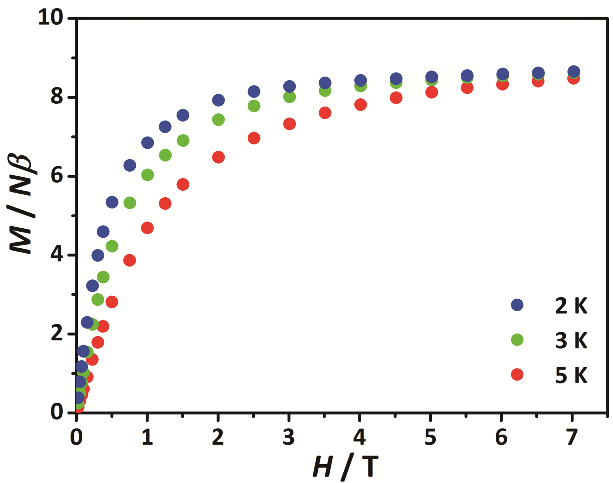

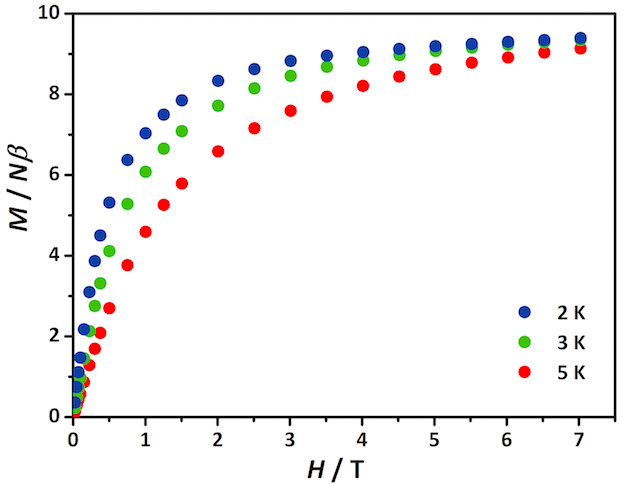

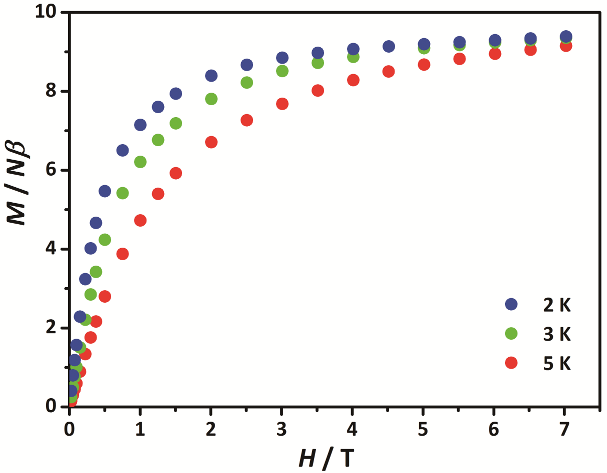
**

**Figure S2 | Field-dependencies of magnetization for** **1·3H_2_O (left), 1·H_2_O (middle) and 1 (right).** The field range is 0-7 T, and the measured temperatures are 2, 3 and 5 K.


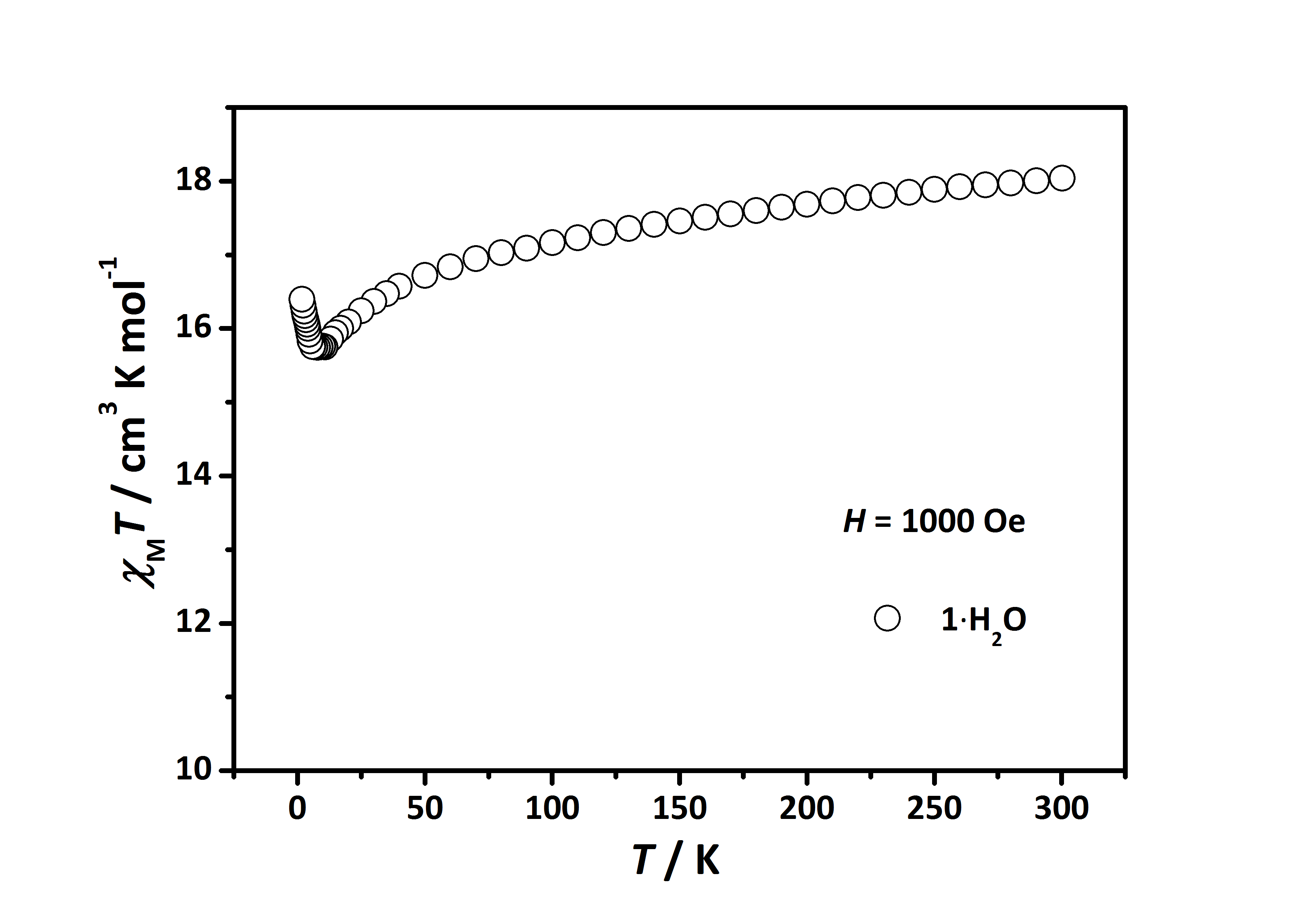


**Figure S3 | Temperature dependencies of *χ*_M_*T* products for 1∙H_2_O.** The temperature range is 1.8 K < *T* < 300 K, and the applied field of 1,000 Oe.


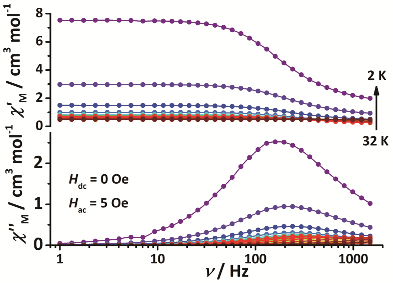

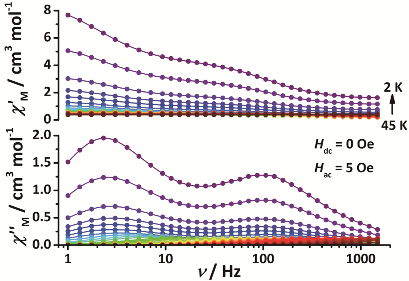

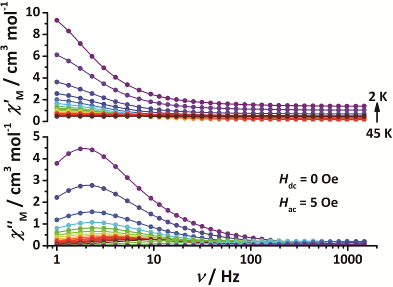


**Figure S4 | Temperature- and frequency-dependencies of the alternating-current susceptibilities for 1·3H_2_O (left), 1·H_2_O (middle) and 1 (right).** Lines are guides to the eyes.


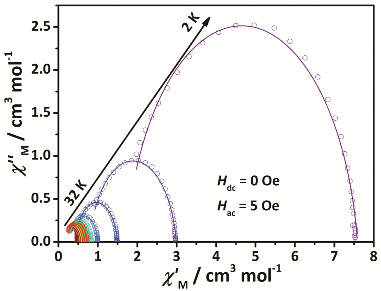

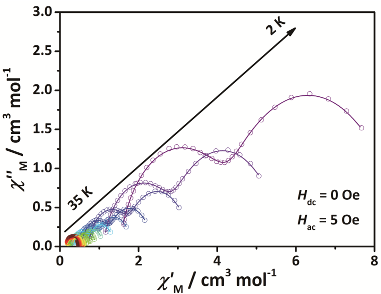

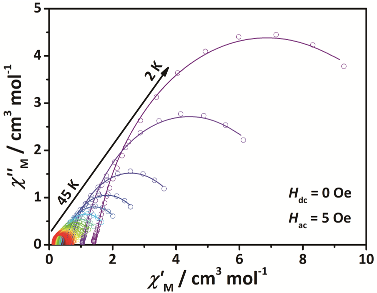


**Figure S5 | Cole-Cole plots for 1·3H_2_O (left), 1·H_2_O (middle) and 1 (right)**. The solid lines are the best fits for the generalized Debye model.


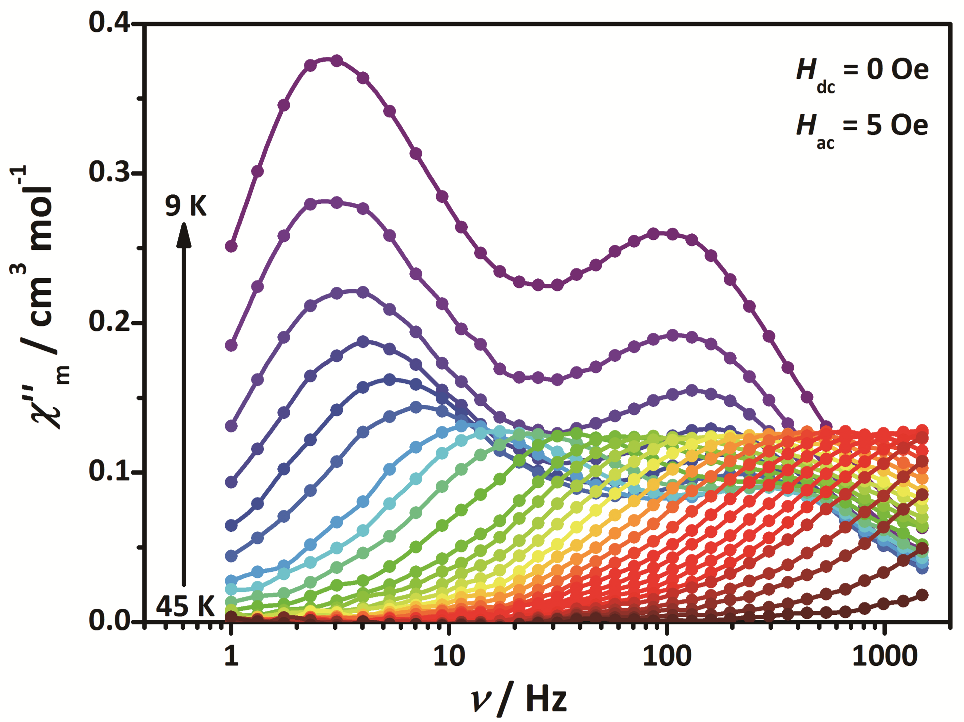


**Figure S6 | Out-of-phase ac magnetic susceptibilities (*χ*_M_'') for 1∙H_2_O.** Lines are guides to the eyes.

**Table S2. Relaxation Fitting Parameters of the Modified Debye Functions**.

| **compound 1∙3H_2_O** | | | | | | | | | | | | | |
| --- | --- | --- | --- | --- | --- | --- | --- | --- | --- | --- | --- | --- | --- |
| **Temperature** | ***τ*** | | | ***α*** | | | | ***χ*_S_** | | | | ***χ*_T_** | |
| **2 K** | 8.34644E-4 | | | 0.09527 | | | | 7.58509 | | | | 1.71575 | |
| **5 K** | 6.72487E-4 | | | 0.09119 | | | | 2.98701 | | | | 0.80132 | |
| **10 K** | 6.29416E-4 | | | 0.0919 | | | | 1.4982 | | | | 0.43188 | |
| **15 K** | 5.99169E-4 | | | 0.08671 | | | | 0.99863 | | | | 0.30187 | |
| **18 K** | 5.5389E-4 | | | 0.08137 | | | | 0.83787 | | | | 0.25673 | |
| **20 K** | 4.99472E-4 | | | 0.07577 | | | | 0.75644 | | | | 0.23271 | |
| **21 K** | 4.64426E-4 | | | 0.0734 | | | | 0.7222 | | | | 0.22193 | |
| **22 K** | 4.2051E-4 | | | 0.06566 | | | | 0.68965 | | | | 0.21235 | |
| **23 K** | 3.64987E-4 | | | 0.04614 | | | | 0.65469 | | | | 0.20584 | |
| **24 K** | 3.04282E-4 | | | 0.05853 | | | | 0.63437 | | | | 0.19127 | |
| **25 K** | 2.36697E-4 | | | 0.05786 | | | | 0.60994 | | | | 0.17897 | |
| **26 K** | 1.76334E-4 | | | 0.05306 | | | | 0.58637 | | | | 0.17074 | |
| **27 K** | 1.19232E-4 | | | 0.06499 | | | | 0.5659 | | | | 0.14789 | |
| **28 K** | 7.93424E-5 | | | 0.07187 | | | | 0.54744 | | | | 0.13361 | |
| **29 K** | 4.65528E-5 | | | 0.07852 | | | | 0.52818 | | | | 0.08822 | |
| **30 K** | 2.42397E-5 | | | 0.09787 | | | | 0.51227 | | | | 0 | |
| **31 K** | 1.72443E-5 | | | 0.06823 | | | | 0.49628 | | | | 0 | |
| **32 K** | 1.20742E-5 | | | 0.05366 | | | | 0.48144 | | | | 0 | |
|  |  | | |  | | | |  | | | |  | |
| **compound 1∙H_2_O** | | | | | | | | | | | | | |
| **Temperature** | ***τ*_1_** | ***τ*_2_** | | | ***α*_1_** | ***α*_2_** | | | ***Δχ*_S1_** | ***Δχ*_S2_** | | | ***χ*_T_** |
| **2 K** | 0.07065 | 0.00143 | | | 0.08884 | 0.12394 | | | 8.56595 | 5.5392 | | | 1.52279 |
| **3 K** | 0.06445 | 0.0014 | | | 0.08552 | 0.1255 | | | 5.3775 | 3.5543 | | | 1.10283 |
| **5 K** | 0.06047 | 0.00141 | | | 0.08373 | 0.13205 | | | 3.06001 | 2.08142 | | | 0.72113 |
| **7 K** | 0.0579 | 0.0014 | | | 0.08265 | 0.13205 | | | 2.12012 | 1.46488 | | | 0.54126 |
| **9 K** | 0.05632 | 0.00137 | | | 0.07976 | 0.13288 | | | 1.61681 | 1.13266 | | | 0.43859 |
| **12 K** | 0.05478 | 0.00123 | | | 0.0824 | 0.13075 | | | 1.23166 | 0.82844 | | | 0.34184 |
| **15 K** | 0.04721 | 0.00106 | | | 0.07451 | 0.12012 | | | 0.95915 | 0.64863 | | | 0.28716 |
| **18 K** | 0.03876 | 9.14315E-4 | | | 0.05389 | 0.09889 | | | 0.78035 | 0.54562 | | | 0.24607 |
| **21 K** | 0.03002 | 7.68204E-4 | | | 0.04701 | 0.09701 | | | 0.66909 | 0.46112 | | | 0.21772 |
| **24 K** | 0.02265 | 6.5346E-4 | | | 0.03953 | 0.0866 | | | 0.58462 | 0.40183 | | | 0.19586 |
| **27 K** | 0.01364 | 5.09935E-4 | | | 0.0391 | 0.08108 | | | 0.52377 | 0.35361 | | | 0.17774 |
| **28 K** | 0.01017 | 4.50946E-4 | | | 0.04288 | 0.08147 | | | 0.50785 | 0.33813 | | | 0.17224 |
| **29 K** | 0.00711 | 3.97718E-4 | | | 0.03622 | 0.07929 | | | 0.4878 | 0.32525 | | | 0.16843 |
| **30 K** | 0.00469 | 3.52053E-4 | | | 0* | 0* | | | 0.46687 | 0.29181 | | | 0.17422 |
| **31 K** | 0.00303 | 2.93497E-4 | | | 0* | 0* | | | 0.44907 | 0.28294 | | | 0.1713 |
| **31.5 K** | 0.00244 | 2.71484E-4 | | | 0* | 0* | | | 0.43472 | 0.28155 | | | 0.1705 |
| **32 K** | 0.00195 | 2.4648E-4 | | | 0* | 0* | | | 0.42244 | 0.28246 | | | 0.16893 |
| **32.5 K** | 0.00153 | 2.11559E-4 | | | 0* | 0* | | | 0.41363 | 0.28143 | | | 0.16568 |
| **33 K** | 0.00123 | 1.94272E-4 | | | 0* | 0* | | | 0.39763 | 0.28256 | | | 0.16588 |
| **33.5 K** | 9.89414E-4 | 1.74205E-4 | | | 0* | 0* | | | 0.38102 | 0.28535 | | | 0.16561 |
| **34 K** | 7.95607E-4 | 1.52457E-4 | | | 0* | 0* | | | 0.36571 | 0.29237 | | | 0.16301 |
| **34.5 K** | 6.40289E-4 | 1.32337E-4 | | | 0* | 0* | | | 0.35094 | 0.29759 | | | 0.16113 |
| **35 K** | 5.29838E-4 | 1.21535E-4 | | | 0* | 0* | | | 0.32427 | 0.31025 | | | 0.16191 |
|  |  | |  | | | |  | | | |  | | |
| **compound 1** | | | | | | | | | | | | | |
| **Temperature** | ***τ*** | | | ***α*** | | | | ***χ*_S_** | | | | ***χ*_T_** | |
| **2 K** | 0.08519 | | | 0.12875 | | | | 12.28399 | | | | 1.44974 | |
| **3 K** | 0.07599 | | | 0.12632 | | | | 7.75649 | | | | 1.07061 | |
| **5 K** | 0.06905 | | | 0.12748 | | | | 4.44433 | | | | 0.70011 | |
| **7 K** | 0.06627 | | | 0.12971 | | | | 3.12243 | | | | 0.52394 | |
| **9 K** | 0.0644 | | | 0.12957 | | | | 2.4147 | | | | 0.42346 | |
| **11 K** | 0.0623 | | | 0.12899 | | | | 1.96846 | | | | 0.35721 | |
| **13 K** | 0.05878 | | | 0.12313 | | | | 1.64371 | | | | 0.30989 | |
| **15 K** | 0.0534 | | | 0.11246 | | | | 1.41683 | | | | 0.27646 | |
| **17 K** | 0.04633 | | | 0.09788 | | | | 1.2396 | | | | 0.25081 | |
| **19 K** | 0.03943 | | | 0.08305 | | | | 1.10539 | | | | 0.22972 | |
| **21 K** | 0.03302 | | | 0.06782 | | | | 0.994 | | | | 0.21322 | |
| **23 K** | 0.02715 | | | 0.0584 | | | | 0.90364 | | | | 0.19788 | |
| **25 K** | 0.02147 | | | 0.04728 | | | | 0.83332 | | | | 0.18677 | |
| **27 K** | 0.0142 | | | 0.03708 | | | | 0.76858 | | | | 0.17507 | |
| **29 K** | 0.00716 | | | 0.03797 | | | | 0.716 | | | | 0.16353 | |
| **31 K** | 0.00281 | | | 0.03925 | | | | 0.67018 | | | | 0.15443 | |
| **33 K** | 0.00102 | | | 0.04934 | | | | 0.63069 | | | | 0.14357 | |
| **35 K** | 0.000373895 | | | 0.04822 | | | | 0.59421 | | | | 0.13374 | |
| **37 K** | 0.00014984 | | | 0.03905 | | | | 0.56094 | | | | 0.12297 | |
| **39 K** | 5.94566E-05 | | | 0.03714 | | | | 0.53282 | | | | 0.08821 | |
|  |  | | |  | | | |  | | | |  | |
| *** The parameters are fixed.** | | | | | | | | | | | | | |

**Compound 1∙3H_2_O and 1 are fitted by**

$\chi_{ac}(\omega)=\chi_{T}+\frac{\chi_{S}-\chi_{T}}{1+(\mathrm{i}\omega\tau)^{1-\alpha}}$;

**Compound 1∙H_2_O is fitted by**

$\chi_{ac}(\omega)=\chi_{T}+\frac{\Delta\chi_{S1}}{1+(\mathrm{i}\omega\tau_{1})^{1-\alpha_{1}}}+\frac{\Delta\chi_{S2}}{1+(\mathrm{i}\omega\tau_{2})^{1-\alpha_{2}}}$.


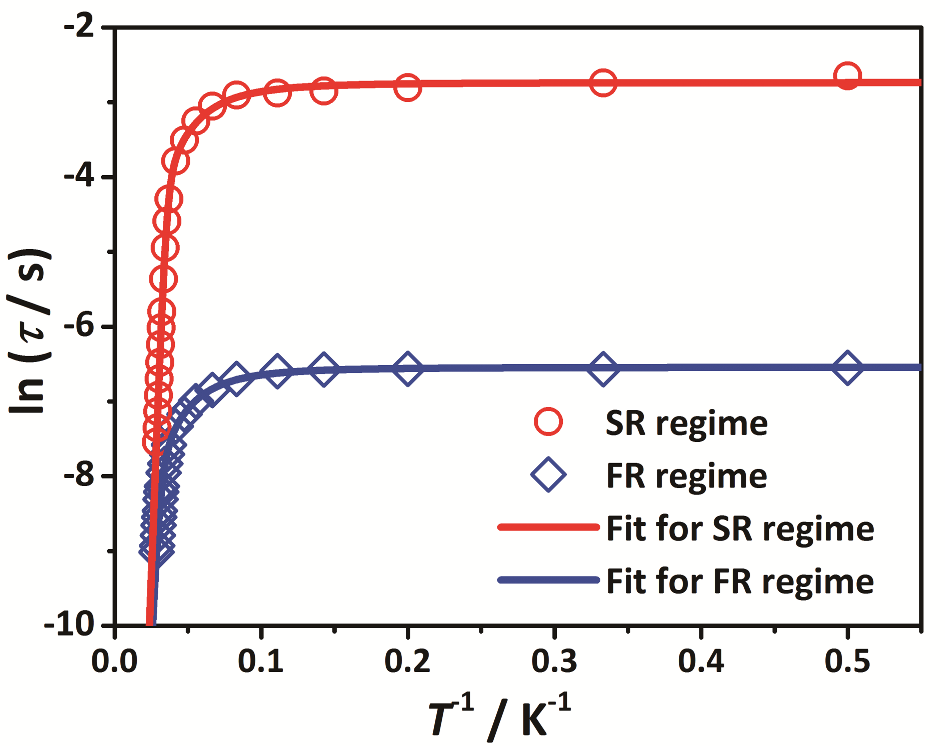


**Figure S7 | Magnetic relaxation dynamics for 1∙H_2_O.** The red and blue solid line respectively represents the best fit for the slow relaxation (SR) regime and fast relaxation (FR) regime.

# 3. Ab initio calculations

### Computational details

All calculations on individual magnetic centers (two Co^2+^ and Dy^3+^) were done with MOLCAS 8.0 and were of CASSCF/RASSI/SINGLE_ANISO type. Calculated structural model for Dy^3+^ fragment was in fact the entire molecular structure, unaltered. Computed fragments of Co^2+^ were smaller, as shown in Figure S8.


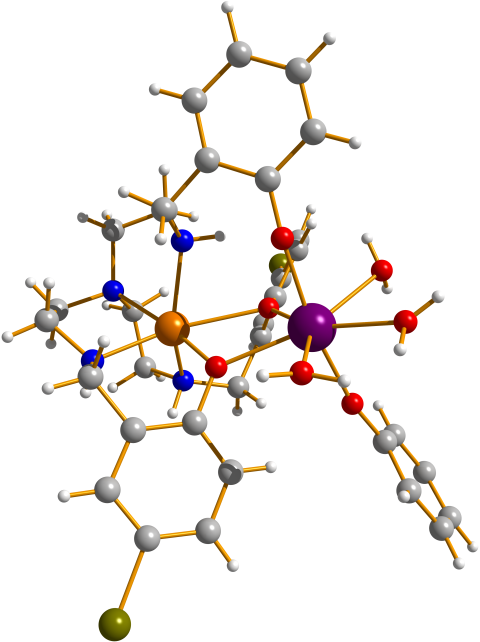


**Figure S8**: Structural model for the Co1 site of **1**. Computed fragment of the Co sites in **1** and **3∙H_2_O** are similar.

Each atom from the fragments was described by an ANO-RCC basis set. Two basis set models were employed: MB (small) and TZP (large).

For Dy^3+^: Active space of the CASSCF included 9 electrons in seven 4*f* orbitals. All spin sextet, spin quartet and spin doublets were optimized within state-average CASSCF calculations. The spin-orbit coupling included the mixing of 21 sextets, 128 quartets and 130 doublet states.

For Co^2+^: Active space of the CASSCF included 7 electrons in five 3*d* +3*d*’ orbitals (second shell added to account for the double shell effect, notable for late 3*d* elements). All spin quartets and spin doublets were optimized within state-average CASSCF calculations. The spin-orbit coupling included the mixing of 10 quartets and 40 doublet spin states.

On the basis of the resulting spin-orbital eigenstates, local magnetic properties of individual sites were computed.

**Table S3**: Energies of the low-lying Kramers doublets (cm^-1^) on the individual magnetic sites in **1**.

| **Dy** | | **Co2** | | **Co1** | |
| --- | --- | --- | --- | --- | --- |
| **MB** | **TZP** | **MB** | **TZP** | **MB** | **TZP** |
| 0.0  0.0  364.6  364.6  406.7  406.7  537.1  537.1  595.8  595.8  692.0  692.0  815.5  815.5  907.0  907.0  **…** | 0.0  0.0  292.5  292.5  327.8  327.8  404.7  404.7  485.5  485.5  543.4  543.4  640.6  640.6  684.9  684.9  … | 0.0  0.0  45.6  45.6  2219.1  2219.1  2336.5  2336.5  3828.3  3828.3  3916.0  3916.0  7122.4  7122.4  7177.6  7177.6  … | 0.0  0.0  44.0  44.0  2315.0  2315.0  2427.5  2427.5  3960.6  3960.6  4045.6  4045.6  7531.4  7531.4  7583.2  7583.2  … | 0.0  0.0  64.5  64.5  1679.6  1679.6  1834.8  1834.8  2746.0  2746.0  2848.9  2848.9  7044.2  7044.2  7108.7  7108.7  … | 0.0  0.0  62.2  62.2  1740.1  1740.1  1884.6  1884.6  2881.8  2881.8  2976.8  2976.8  7496.9  7496.9  7556.4  7556.4  … |
| **main values of the *g* tensor in the ground and first excited Kramers doublets on metal sites** | | | | | |
| 0.00013  0.00017  19.92132 | 0.00178  0.00230  19.86148 | 1.4212  1.9573  6.8624 | 1.5336  2.1941  6.6798 | 1.5655  2.3084  7.0063 | 1.6347  2.4741  6.7994 |
| 0.05881  0.10390  16.83619 | 0.09458  0.52696  18.68469 | 1.9693  2.5339  5.6879 | 1.8313  2.3106  5.7932 | 1.8931  2.2767  5.7579 | 1.7642  2.1246  5.8380 |


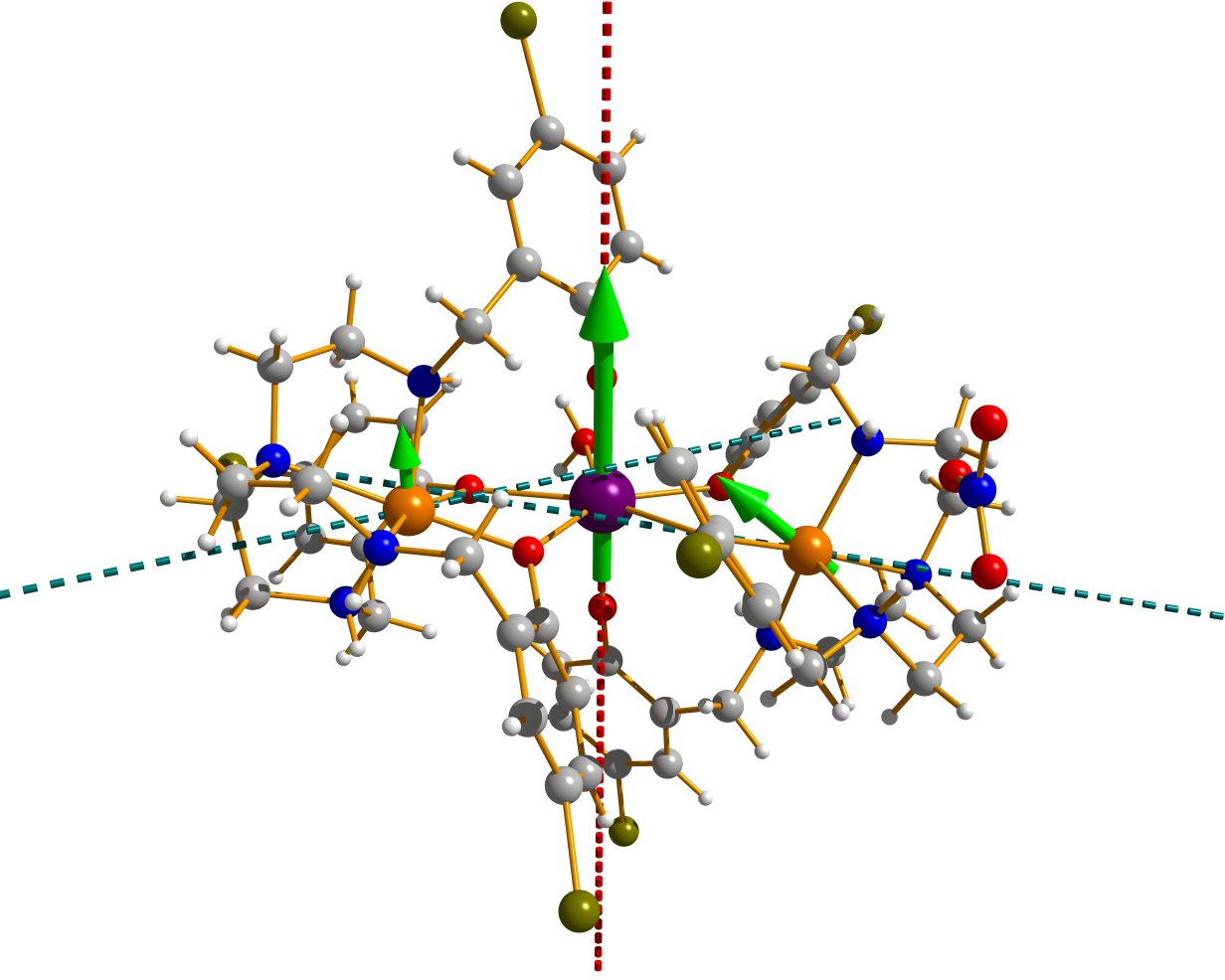


**Figure S9**: Orientation of the local main anisotropy axes on Dy and Co sites (*g*_Z_ of the ground Kramers doublet in Table S3, dashed line) and local magnetic moments (green arrows) on metal sites in the ground exchange state of **1**.

**Table S4**: Energies of the low-lying Kramers doublets (cm^-1^) on the individual magnetic sites in **1∙3H_2_O**.

| **Dy** | | **Co1** | | **Co2** | |
| --- | --- | --- | --- | --- | --- |
| **MB** | **TZP** | **MB** | **TZP** | **MB** | **TZP** |
| 0.0  0.0  351.5  351.5  459.5  459.5  560.5  560.5  598.1  598.1  668.6  668.6  749.0  749.0  788.0  788.0  **…** | 0.0  0.0  294.5  294.5  313.2  313.2  404.2  404.2  466.5  466.5  494.9  494.9  572.0  572.0  599.3  599.3  … | 0.0  0.0  95.6  95.6  1146.6  1146.6  1357.3  1357.3  2018.0  2018.0  2137.3  2137.3  6883.2  6883.2  6949.6  6949.6  … | 0.0  0.0  93.8  93.8  1156.1  1156.1  1354.0  1354.0  2097.6  2097.6  2206.4  2206.4  7326.1  7326.1  7388.5  7388.5  … | 0.0  0.0  131.7  131.7  886.3  886.3  1112.6  1112.6  1931.1  1931.1  2028.8  2028.8  8099.3  8099.3  8152.1  8152.1  … | 0.0  0.0  128.5  128.5  898.1  898.1  1113.1  1113.1  1995.9  1995.9  2087.1  2087.1  8591.8  8591.8  8637.9  8637.9  … |
| **main values of the *g* tensor in the ground and first excited Kramers doublets on metal sites** | | | | | |
| 0.00008  0.00009  19.85041 | 0.00141  0.00169  19.85877 | 1.5381  2.2145  7.3064 | 1.5676  2.3017  7.2502 | 1.4663  2.0481  7.7200 | 1.4828  2.1311  7.6393 |
| 0.01428  0.02934  16.89706 | 0.71248  3.81795  15.28559 | 2.0874  2.4598  5.6064 | 2.0298  2.3654  5.6333 | 2.3930  2.5679  5.2923 | 2.3311  2.5138  5.3244 |


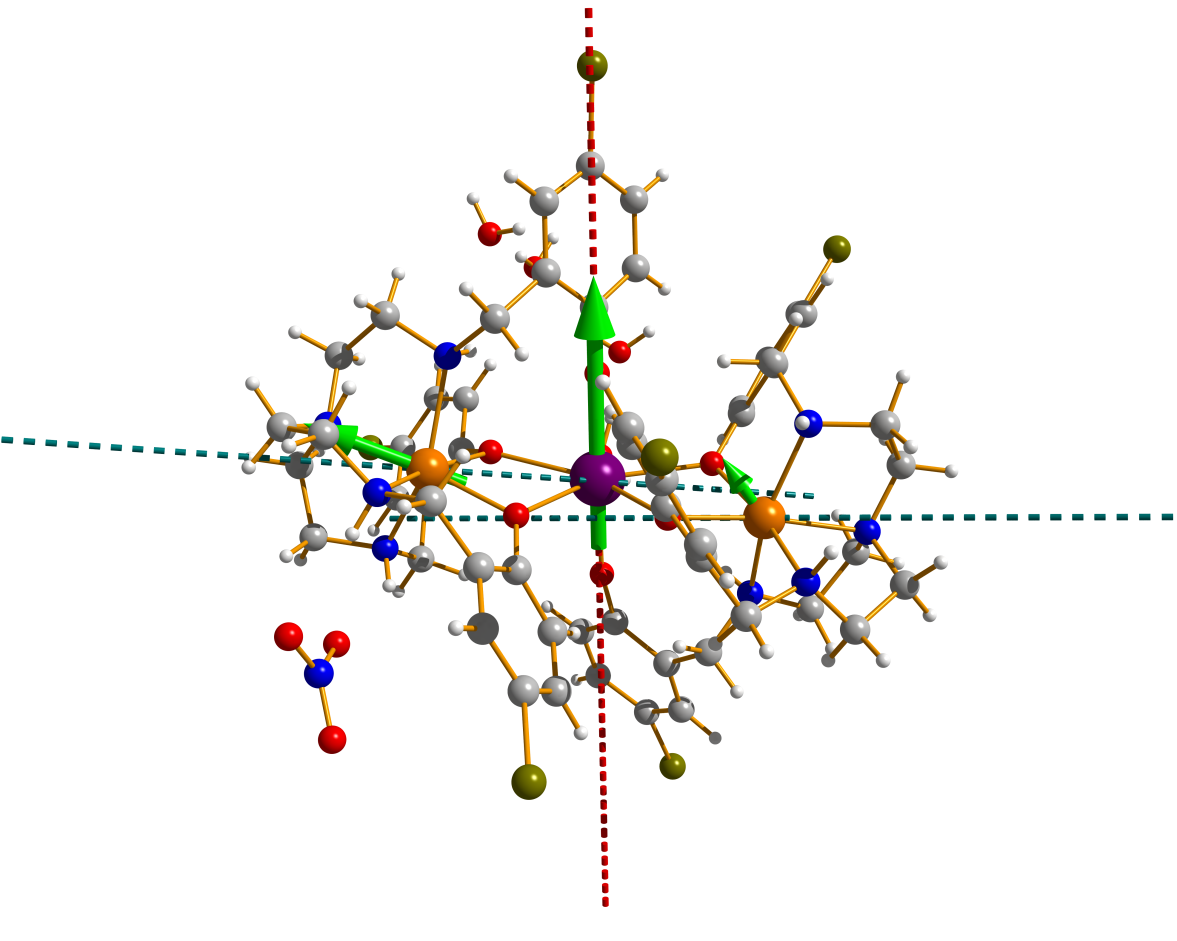


**Figure S10**: Orientation of the local main anisotropy axes on Dy and Co sites (*g*_Z_ of the ground Kramers doublet in Table S4, dashed line) and local magnetic moments (green arrows) on metal sites in the ground exchange state of **1∙3H_2_O**.

**Table S5*.*** Angle of the main magnetic axis on the Dy sites (Tables S3, and S4) with the shortest O1--O4 axis of the closest two oxygen atoms displaying strongest axial perturbation (in degrees)

|  | **1** | | **1∙3H_2_O** | |
| --- | --- | --- | --- | --- |
| basis | **MB** | **TZP** | **MB** | **TZP** |
| axial Dy-O1  axial Dy-O4 | 2.17578  2.17156 | | 2.17614  2.19806 | |
| avg. length of five non-axial Dy-O bonds | 2.37896 | | 2.38729 | |
| angle | 1.926 | 0.736 | 1.516 | 0.981 |

### Ab initio calculated parameters of the crystal-field for Dy^3+^ sites

Recently, the extraction of the parameters of the multiplet-specific crystal-field for lanthanides methodology has been implemented in the SINGLE_ANISO program in MOLCAS. The results presented below use the ab initio CASSCF/RASSI wave function and energies to compute the parameters of the crystal-field splitting of the ground *J* multiplet.

The Crystal-Field Hamiltonian:


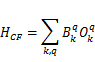


where:


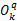
 -- Extended Stevens Operators (ESO)as defined in:

1. Rudowicz, C.; J. Phys. C: Solid State Phys.,18 (1985) 1415-1430.

2. Implemented in the "EasySpin" function in MATLAB, www.easyspin.org.


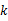
 - the rank of the ITO, = 2, 4, 6.


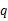
 - the component (projection) of the ITO, =
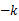
,
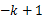
, ...
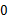
,
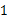
, ...
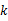
;

Quantization axis was chosen the main magnetic axis of the ground Kramers doublet.

Table S6*.* Parameters of the Crystal-Field acting on the ground atomic multiplet *J* = 15/2 for the investigated compounds (TZP basis set).

| ***k*** | ***q*** | **1** | **1∙3H_2_O** |
| --- | --- | --- | --- |
| **2** | -2  -1  0  1  2 | -0.16952862E+01  -0.66548425E+00  **-0.21717037E+01**  -0.74679831E+00  **0.25592847E+01** | -0.21479543E+00  0.72677092E+00  **-0.19541131E+01**  0.36404451E+00  **0.19958333E+01** |
| **4** | -4  -3  -2  -1  0  1  2  3  4 | **-0.11317237E-01**  -0.76638136E-02  0.18694246E-02  0.53635590E-02  **-0.12326977E-01**  0.61765138E-02  -0.13824500E-02  0.18501540E-02  -0.11704870E-02 | -0.30224804E-02  **0.30974082E-01**  -0.54865382E-04  -0.38187018E-02  **-0.11871038E-01**  -0.50983570E-02  -0.75514578E-03  -0.23844795E-02  0.16263973E-02 |
| **6** | -6  -5  -4  -3  -2  -1  0  1  2  3  4  5  6 | **0.99438871E-04**  0.59093552E-05  -0.32161327E-04  -0.53657461E-04  -0.19346586E-04  -0.25030334E-04  0.96327799E-05  -0.24280777E-04  -0.93829392E-05  -0.28101726E-04  -0.12733710E-04  **-0.12240275E-03**  -0.51329013E-04 | **0.11240136E-03**  0.67210529E-04  0.44982418E-05  **0.21617012E-03**  -0.26643304E-04  0.63187342E-05  0.96708283E-05  0.43168332E-04  0.21323567E-04  0.87261364E-05  0.15771717E-04  0.62856110E-04  -0.11677186E-04 |

### Calculated atomic charges

**Table S7*.*** Calculated LoProp atomic charges on the calculated structures for **1** (using the TZP calculation results). Atoms from the first coordination sphere of Dy are highlighted.

| **Atom label** | **Cartesian coordinates (Angstrom)** | **LoProp charge** |
| --- | --- | --- |
| Dy1  Co2  Co1  Br1  Br2  Br3  Br4  Br5  Br6  **O1**  O2  O3  **O4**  O5  O6  O1W  O7  O8  O9  N1  N2  N3  N4  N5  N6  N7  N8  N9  C1  C2  C3  C4  C5  C6  C7  C8  C9  C10  C11  C12  C14  C13  C15  C16  C17  C18  C19  C20  C21  C22  C23  C24  C25  C26  C27  C28  C29  C30  C31  C32  C33  C34  C35  C36  C37  C38  C39  C40  C41  C42  C43  C44  C45  C46  C47  C48  C49  C50  C51  C52  C53  C54  H1WA  H1WB  H2N  H3N  H4N  H6N  H7N  H8N  H2  H3  H5  H7A  H7B  H8A  H8B  H9A  H9B  H11  H12  H14  H16A  H16B  H17A  H17B  H18A  H18B  H20  H21  H23  H25A  H25B  H26A  H26B  H27A  H27B  H29  H30  H32  H34A  H34B  H35A  H35B  H36A  H36B  H38A  H39  H41  H43A  H43B  H44A  H44B  H45A  H45B  H47  H48  H50  H52A  H52B  H53A  H53B  H54A  H54B | 10.82343400 -1.43305500 41.75232700  8.10426600 0.49894500 43.22337900  13.94186700 -2.87902800 42.79494700  8.69942200 -9.30896500 41.72086900  12.91978800 1.93280800 48.27431500  16.08022700 -3.14222600 35.96212100  11.88647900 5.70154100 38.05896900  3.66222500 -0.97528500 37.94362200  9.62646800 -2.87418400 49.30457500  10.14177000 -3.48292900 42.01185900  12.54360400 -1.57595700 43.39752400  12.73699900 -2.46889200 40.91456200  11.48721300 0.53446900 41.11679400  8.64868200 -0.76214300 41.27034100  9.72586800 -0.64265500 43.66716600  10.48281000 -1.81170500 39.29670500  16.54666100 -0.91069600 46.83547000  16.53834300 -0.73469300 44.70828800  17.10604900 0.93976100 45.91044500  15.57345000 -4.33549600 42.43879300  12.84305400 -4.76178000 42.47624400  14.65222700 -3.03242500 44.77944300  15.41956700 -1.61471000 41.97815300  6.41528800 2.02807600 43.07170500  8.84623600 1.86337500 41.79839100  6.48391200 -0.79605200 43.39377800  8.06018100 1.29984200 45.17267200  16.73173700 -0.22928900 45.81681900  9.81528700 -4.77146800 41.92946800  8.73186200 -5.18321900 41.18046000  8.41577700 -6.50728100 41.12053900  9.15400300 -7.46641900 41.76843100  10.22495100 -7.03367700 42.55114400  10.58262600 -5.70961500 42.64851600  11.79706000 -5.26072500 43.40126900  13.70605000 -5.86139700 42.09050500  14.98079000 -5.50616100 41.70476500  12.77443000 -0.72177500 44.44613500  12.59143300 0.62489300 44.34876400  12.66213600 1.44678000 45.49849100  13.30054600 -0.44888900 46.81674500  12.97198300 0.83642000 46.67443400  13.20904800 -1.29661200 45.64080200  13.57504100 -2.75953900 45.76813400  15.21993400 -4.42592000 44.90303000  16.17650800 -4.63421800 43.74581200  13.45858900 -2.54155400 39.80603000  13.06556100 -3.44579100 38.74992900  13.79131000 -3.58788600 37.56275100  15.03693700 -2.85157800 37.49159500  15.41332800 -2.00062600 38.51024600  14.62103500 -1.80363100 39.66746400  15.02238000 -0.88001700 40.73854500  16.74629400 -2.27028200 41.85456700  16.56329700 -3.67508000 41.53623900  11.62862000 1.69060100 40.46890200  12.43547000 1.78264000 39.30045000  12.53528600 2.95007500 38.62259800  11.80121900 4.08198700 39.01208200  11.04011900 4.02385700 40.19551400  10.94030200 2.88064300 40.90707200  10.03571500 2.73208900 42.09799500  7.77528800 2.59645400 41.11679400  6.83534900 3.12284900 42.12046500  7.60684800 -0.86709900 40.49137200  7.63596100 -0.43597200 39.16562800  6.45895800 -0.45373400 38.36419000  5.27779600 -0.99627600 38.95216100  5.26739900 -1.50006600 40.24045500  6.42360600 -1.47746000 41.01567800  6.46519700 -1.93926700 42.44628300  5.17798000 -0.03067900 43.46493400  5.26531900 1.23525300 42.67473100  9.74250400 -1.14321500 44.94797000  9.81112800 -2.49634200 45.15769200  9.78201500 -3.00497500 46.46096600  9.70715300 -2.12172900 47.52455800  9.62397200 -0.76537300 47.38224600  9.62189300 -0.27450100 46.04152200  9.35363600 1.19488500 45.82430900  7.54862100 2.69333600 45.13896700  6.23437100 2.65296900 44.40868400  10.31229000 -1.06570900 38.92220100  9.83192300 -2.34294400 39.16937300  12.41883400 -4.65843800 41.68604000  15.32183000 -2.44305600 44.89554000  15.51938300 -0.95752300 42.58485000  9.15608300 1.34020900 41.13551900  6.58788800 -1.15290300 44.21394200  7.45712300 0.83803400 45.65952800  8.20782600 -4.54540900 40.71233000  7.65883500 -6.77532300 40.61121400  10.72819200 -7.67956100 43.03425500  11.54544000 -4.54056500 44.03418000  12.15681500 -6.01802400 43.92931900  13.77259500 -6.48467500 42.85823800  13.27559200 -6.34742500 41.34524200  14.96831300 -5.29463400 40.73854500  15.57553000 -6.28768100 41.83209700  12.41675400 1.01726700 43.49864000  12.50201400 2.38331200 45.44980500  13.58751800 -0.79443700 47.65188900  13.89112700 -2.95653400 46.68192400  12.78690700 -3.32791700 45.57713700  14.49002600 -5.09441000 44.86557900  15.69614100 -4.52603200 45.76438900  16.96880200 -4.05453700 43.87688900  16.48635500 -5.57559400 43.74955700  12.27950700 -3.96572800 38.86977000  13.48562200 -4.13365800 36.85119400  16.23473500 -1.52913000 38.43160000  14.26959800 -0.27127100 40.94826700  15.78348100 -0.32778600 40.42770700  17.27241000 -1.83108100 41.13926400  17.24329700 -2.17985800 42.70469100  17.43253200 -4.14011600 41.61113900  16.25345000 -3.75904500 40.59997900  12.91167800 1.01888200 38.99710200  13.11131100 3.00336100 37.86984400  10.58886500 4.80376200 40.50260800  10.54727500 2.33648500 42.84700300  9.72170900 3.62663900 42.38261800  7.29908000 1.99093700 40.49137200  8.16207700 3.34245000 40.59248900  6.03889700 3.49100300 41.66357000  7.26788700 3.85592700 42.62604500  8.45112900 -0.12433300 38.79112400  6.45895800 -0.11948900 37.47661500  4.46470800 -1.86337500 40.59997900  5.67290300 -2.50280000 42.63353500  7.27204600 -2.49311200 42.58859500  4.44599200 -0.59259900 43.10541000  4.96794900 0.18084800 44.40868400  5.33394300 1.01726700 41.71225600  4.43975400 1.76487800 42.80955200  9.87559300 -3.09055500 44.41991900  9.81528700 -3.94150700 46.61451300  9.57198500 -0.18246200 48.13499900  9.34323800 1.67445400 46.68941400  10.05859000 1.59210400 45.25506300  8.18703100 3.28432000 44.66709200  7.42177100 3.03727000 46.06024700  5.57724600 2.12980200 44.93299000  5.88293400 3.57173900 44.30382300 | 2.5644  0.6085  1.7155  -0.0460  -0.0785  -0.0234  -0.0508  -0.0757  -0.0944  **-0.9382**  -0.8915  -0.8791  **-0.9409**  -0.8656  -0.8941  -0.7450  -0.5521  -0.5967  -0.4988  -0.3086  -0.4107  -0.3829  -0.4026  -0.2672  -0.3529  -0.3613  -0.3515  0.6943  0.3070  -0.2195  -0.1105  -0.0948  -0.1388  -0.1115  -0.1190  -0.1240  -0.1167  0.2459  -0.1669  -0.1233  -0.1192  -0.0152  -0.0451  -0.1200  -0.1281  -0.1281  0.2724  -0.1972  -0.1295  -0.0813  -0.1564  -0.0875  -0.1245  -0.1184  -0.1167  0.2978  -0.2390  -0.1301  -0.0988  -0.1404  -0.0848  -0.1399  -0.1301  -0.1268  0.3068  -0.2632  -0.1332  -0.0977  -0.1508  -0.0763  -0.1320  -0.1372  -0.1250  0.2721  -0.2013  -0.1604  -0.0515  -0.1515  -0.0451  -0.1252  -0.1330  -0.1332  0.3801  0.3819  0.2640  0.2637  0.2620  0.2349  0.2057  0.1925  0.1613  0.1638  0.1501  0.1522  0.1266  0.1239  0.1468  0.1433  0.1364  0.1684  0.1678  0.1765  0.1509  0.1401  0.1301  0.1565  0.1468  0.1351  0.1568  0.1705  0.1578  0.1729  0.1397  0.1427  0.1612  0.1331  0.1462  0.1433  0.1583  0.1529  0.1411  0.1155  0.1342  0.1136  0.1159  0.1182  0.1299  0.1619  0.1530  0.1113  0.1532  0.1210  0.1210  0.1378  0.1071  0.1530  0.1552  0.1628  0.1115  0.1374  0.1253  0.1143  0.1161  0.1155 |

**Table S8*.*** Calculated LoProp atomic charges on the calculated structure of **1∙3H_2_O** (using the TZP calculation results). Atoms from the first coordination sphere of Dy are highlighted.

| **Atom label** | **Cartesian coordinates ( Angstrom)** | **LoProp charge** |
| --- | --- | --- |
| Dy1  Co1  Co2  Br1  Br2  Br3  Br4  Br5  Br6  **O1**  O2  O3  **O4**  O5  O6  O1W  O7  O8  O9  O2W  O3W  O4W  N8  N1  N2  N3  N4  N5  N6  N7  N9  C1  C2  C3  C4  C5  C6  C7  C8  C9  C10  C11  C12  C13  C14  C15  C16  C17  C18  C19  C20  C21  C22  C23  C24  C25  C26  C27  C28  C29  C30  C31  C32  C33  C34  C35  C36  C37  C38  C39  C40  C41  C42  C43  C44  C45  C46  C47  C48  C49  C50  C51  C52  C53  C54  H1WA  H1WB  H2N  H3N  H4N  H6N  H7N  H8N  H2  H3  H5  H7A  H7B  H8A  H8B  H9A  H9B  H11  H12  H14  H16A  H16B  H17A  H17B  H18A  H18B  H20  H21  H23  H25A  H25B  H26A  H26B  H27A  H27B  H29  H30  H32  H34A  H34B  H35A  H35B  H36A  H36B  H38  H39  H41  H43A  H43B  H44A  H44B  H45A  H45B  H47  H48  H50  H52A  H52B  H53A  H53B  H54A  H54B  H2WA  H2WB  H3WA  H3WB  H4WA  H4WB | 10.15220300 8.01613700 4.31123100  7.19791700 9.49023500 5.59545700  13.11310100 6.38484400 5.45993800  12.81340500 15.75241000 4.29454000  4.72249100 9.60192600 -1.17204400  8.00109200 4.47639100 10.87445600  9.15594300 0.63864300 1.05018700  12.04002500 9.20368900 11.81238600  16.39713900 10.48304400 0.39363500  10.88517400 10.05855500 4.47511000  8.18617300 9.17393700 3.72735100  8.61462000 8.12862300 6.11095000  9.49543900 5.99185700 3.76132300  11.37886800 7.14695200 6.16818600  12.42280000 7.44924900 3.76686200  10.11744900 8.08248300 1.89284700  4.74554500 7.13104100 7.46208700  4.51283500 5.82320600 9.15405500  4.55415700 7.93610800 9.43137200  11.12005900 9.89786000 0.02954100  9.91953600 12.30192100 -0.45050200  9.52153700 13.35996300 2.07157100  14.50849100 7.94724500 5.90637800  5.57416600 10.93840000 5.31592500  8.32101400 11.44116900 5.38054600  5.65898600 8.16044400 4.87982400  6.56155300 9.63215600 7.59871500  14.93041400 5.33634800 4.88499300  12.29883300 4.73334400 4.29047800  13.49718100 5.36021400 7.28484000  4.58460500 6.95920900 8.66551800  11.26577500 11.31547600 4.39054900  12.26186100 11.72437400 3.48585200  12.68813300 13.03380000 3.45261800  12.12701900 13.95501200 4.29084800  11.11136000 13.61134700 5.17708100  10.67856300 12.31146800 5.22139300  9.55198500 11.85165700 6.11870400  7.40974800 12.58512600 5.18446700  6.12223100 12.12213400 4.58625900  7.42497200 9.26939900 2.62842100  7.72945200 10.18106500 1.61257600  6.92257900 10.31789400 0.49961400  5.78947700 9.52078300 0.40508300  5.46107300 8.60593500 1.38510900  6.28099600 8.48978900 2.49474800  5.97651500 7.49698000 3.57927600  4.35841900 8.85572900 4.88167000  4.51935900 10.29880200 4.49394300  8.43193200 7.31242000 7.15707500  8.64506900 5.93457900 7.04629600  8.50805200 5.09928300 8.14116300  8.17094900 5.62114400 9.36453500  7.90779100 6.96557300 9.49414700  8.01435900 7.82155300 8.41257200  7.65115700 9.28053600 8.54255300  6.05698500 10.99567700 7.78408500  5.06524900 11.29956600 6.66521500  9.33667400 4.80016800 3.21333500  8.42975700 4.57901300 2.16868700  8.34493800 3.34595700 1.54352400  9.14963600 2.32450900 1.97187000  10.01305500 2.46770300 3.05012100  10.12397300 3.70553200 3.66753000  11.12005900 3.95850800 4.75612000  13.34276600 3.86781800 3.68340800  14.61505900 4.68720400 3.58149200  11.52240800 7.59880700 7.44547000  11.34624500 8.95119100 7.75454400  11.48761100 9.43645800 9.03589000  11.81818900 8.55024900 10.03918000  12.04872400 7.21377500 9.76777100  11.90300900 6.71737100 8.47460900  12.28360900 5.31884700 8.13008500  14.07569400 4.07942700 6.89415900  15.25881800 4.34831200 5.96915300  13.30796800 8.12225900 3.00580900  13.32319200 8.02043300 1.63731700  14.20401100 8.73321900 0.83822900  15.13050100 9.52555600 1.45490000  15.18487300 9.64170200 2.82487000  14.27578100 8.95278200 3.63134200  14.25185800 9.18666500 5.11467600  15.88517700 7.42379300 5.80483000  15.98957100 6.35779600 4.71845500  10.56982000 8.73640100 1.58783500  10.40235500 7.35696900 1.55460100  8.64506900 11.33297800 4.54933200  5.61548900 7.52084600 5.51311200  5.87647200 9.06733700 7.75454400  11.92910700 5.15178800 3.58555300  14.11701600 5.84866300 7.71761800  14.39757300 8.14453400 6.77599400  12.64681100 11.08954900 2.89503000  13.37321400 13.29632100 2.84702500  10.72423500 14.26685600 5.74205500  9.86951500 11.08636700 6.66152300  9.32145000 12.58512600 6.74276100  7.83602100 13.25018100 4.58995200  7.23358400 13.02266200 6.05593000  5.46107300 12.85878500 4.61580000  6.27012100 11.88188700 3.63725000  8.50805200 10.71883600 1.68753600  7.13571600 10.93999100 -0.18463200  4.68464800 8.06657300 1.29980900  5.20661500 6.94011600 3.30122000  6.75729000 6.89874900 3.70002500  3.95607000 8.80004200 5.78636700  3.74510900 8.41023700 4.24653600  4.76076900 10.36085200 3.53754900  3.66028900 10.77611400 4.62687800  8.88647800 5.56545800 6.20363500  8.64941800 4.16534300 8.04626300  7.64680800 7.31242000 10.33939200  7.35972600 9.46827900 9.47162200  8.44715600 9.83899100 8.35275200  6.80731200 11.64164000 7.75454400  5.61113900 11.07682000 8.66293300  4.22575300 10.79998000 6.83876900  4.84776300 12.26532700 6.68367800  7.86211900 5.28543500 1.88324600  7.73597700 3.21071900 0.82715100  10.52197300 1.72786900 3.36030200  10.67638800 4.45491200 5.49095600  11.42671400 3.09139100 5.12169200  13.05785900 3.56711200 2.78425100  13.49500600 3.06911600 4.24653600  15.36538600 4.10011000 3.31599100  14.50631600 5.38089700 2.88395200  11.12440900 9.55578600 7.05663500  11.35711900 10.35926100 9.22421500  12.30970800 6.62827300 10.46863400  12.46412300 4.80335000 8.95465200  11.54415700 4.87653800 7.64007200  13.39713800 3.52733600 6.42888600  14.37799900 3.58620400 7.69915400  16.01566900 4.69356800 6.50273900  15.54372400 3.49551500 5.55373100  12.70118200 7.43493000 1.21857100  14.16703800 8.67116800 -0.10708700  15.84603000 10.19538400 3.22367500  14.93476400 9.86444800 5.34694300  13.36669000 9.55419500 5.36909900  16.15268500 7.03398800 6.67629300  16.50501300 8.16680800 5.59804200  15.90257600 6.78419500 3.82926800  16.87908800 5.92344200 4.76350600  10.57851900 10.51200100 -0.19571000  11.90953300 10.13015200 0.24371400  9.78687000 12.65035900 0.31387400  10.48717500 12.76809600 -0.87515600  9.41931900 14.17775700 1.86478300  10.19356900 13.25813600 2.58115500 | 2.5638  1.7080  0.6067  -0.0651  -0.0454  -0.0394  -0.0851  -0.0764  -0.0789  **-0.9127**  -0.8861  -0.8804  **-0.9434**  -0.8723  -0.8757  -0.7638  -0.5815  -0.4968  -0.5666  -0.6252  -0.5968  -0.5983  -0.3492  -0.3034  -0.4103  -0.3871  -0.3780  -0.2516  -0.3476  -0.3565  0.6917  0.3041  -0.2200  -0.1348  -0.0941  -0.1506  -0.1075  -0.1244  -0.1259  -0.1216  0.2592  -0.1862  -0.1265  -0.0572  -0.1537  -0.0688  -0.1264  -0.1220  -0.1221  0.2576  -0.1710  -0.1238  -0.0378  -0.1262  -0.0590  -0.1251  -0.1253  -0.1307  0.2924  -0.2334  -0.1360  -0.0652  -0.1479  -0.0789  -0.1377  -0.1425  -0.1277  0.2936  -0.2258  -0.1500  -0.0807  -0.1465  -0.0578  -0.1261  -0.1259  -0.1408  0.2834  -0.2291  -0.1537  -0.0744  -0.1636  -0.0492  -0.1331  -0.1413  -0.1306  0.3743  0.3836  0.2569  0.2652  0.2604  0.2240  0.1969  0.2044  0.1615  0.1686  0.1601  0.1562  0.1273  0.1450  0.1264  0.1333  0.1496  0.1579  0.1745  0.1623  0.1380  0.1714  0.1598  0.1406  0.1483  0.1318  0.1650  0.1636  0.1680  0.1471  0.1369  0.1284  0.1563  0.1466  0.1339  0.1435  0.1612  0.1558  0.1422  0.1149  0.1212  0.1126  0.1081  0.1331  0.1356  0.1501  0.1574  0.1161  0.1415  0.1279  0.1136  0.1185  0.1175  0.1428  0.1616  0.1544  0.1123  0.1515  0.1177  0.1145  0.1336  0.1075  0.3256  0.3138  0.3107  0.2880  0.2920  0.3307 |

### Modelling of the exchange interactions

# Estimation of the exchange interactions by BS-DFT calculation

The calculations were carried out on the experimental geometry, by substituting computationally the Dy with Gd. This substitution is necessary in order to avoid the near-degenerate ground state of the Dy^III^ ion (it is known that DFT methods are not appropriate for description of near-degenerate ground states). We have employed ORCA program package for these calculations [1].

Computational details: B3LYP functional, SVP basis sets on all atoms, TightSCF convergence thresholds, Grid6.

Further, the obtained energies of the high-spin state, and of the three low-spin states, corresponding to spin flip of one metal site, were used for the estimation of the exchange coupling constants. General equations derived from the [2] were employed for this case.

The obtained exchange parameters corresponding to Gd-Fe, were re-scaled for the interacting pair of Dy-Fe, by using the rescaling factor 7/5 (the spin of Gd) / (the spin of Dy). This approach was successfully used in [3] and [4].

# Estimation of the exchange interactions by BS-DFT calculation

Localization of the magnetic orbitals observed in transition metal complexes and networks [5] is even more pronounced in lanthanide-containing mono- and polynuclear compounds. This fact allows treating electronic and magnetic structure of polynuclear compounds in a two-step procedure. In the first step, reasonable fragmentation of the cluster into mononuclear fragments is performed and reliable ab initio calculations for each fragment are done. In the second step, the magnetic interaction between the fragments is introduced in an effective way. The exchange interaction between magnetic centers is considered within the Lines model [5], while the account of the dipole-dipole magnetic coupling is treated exactly. The Lines model [6] is an approximation allowing describing the anisotropic exchange interaction between magnetic sites via a single parameter. To this end, an isotropic Heisenberg model involving the true spins of the two centers is introduced with an effective parameter (the Lines exchange parameter). In a second step the matrix of this model is constructed in the basis of products of localized lowest states of the two centers obtained in fragment ab initio calculations of corresponding centers with spin-orbit coupling included. The obtained exchange matrix describes exactly the anisotropic exchange interaction in two limiting cases:

1) of two strongly axial doublets on the two sites (the case of extreme magnetic anisotropy)

2) of isotropic spins on the two sites (lack of magnetic anisotropy)

The Lines approximation is expected to be quite accurate for the description of the exchange interaction between a strongly axial doublet and an arbitrary isotropic spin. For all other cases, the Lines model [6] is a reasonable approximation. Efficient implementation of the Lines model was done in the program POLY_ANISO.

The dipolar magnetic coupling is computed exactly, while the exchange parameters are fitted (from comparison of computed and measured magnetic susceptibility and molar magnetization). Best-fitted values are given in Table 5.

**Table S9*.*** Exchange energy spectrum (Kramers doublets) of the investigated compounds using the exchange coupling constants reported in Table 5 (in cm^-1^). Low-lying exchange states are highlighted.

| **1** | | **1∙3H_2_O** | |
| --- | --- | --- | --- |
| **Lines** | **BS-DFT** | **Lines** | **BS-DFT** |
| 0.000000  0.789273  1.262831  2.280309  43.980828  44.950320  45.280014  46.367752  62.418392  63.145431  63.505175  64.350108  106.387955  107.115686  107.663933  108.501825  289.914013  293.132369  293.648862  297.682258  328.100801  328.669799  329.117558  329.923278  335.290955  336.340467  338.845266  340.083741  353.280283  354.283422  357.199063  358.661170  372.084925  372.814907  373.126896  373.976791  390.470605  391.010581  391.351349  392.016501  398.729734  399.617636  400.102121  401.157881  402.785322  405.394188  405.860955  409.316111  434.436845  434.967553  435.494641  436.144870  447.898729  448.766286  450.864420  451.937595  465.934687  466.735357  469.194461  470.467505  485.104824  486.060330  486.756067  488.661196  511.116813  511.821549  512.303533  513.201676  529.478848  529.834123  531.077692  532.263184  543.312070  543.715309  545.052409  545.992160  547.882700  548.154995  549.403952  550.387077  586.748632  588.229376  588.580263  590.516753  592.297086  592.524148  592.788086  594.285526  605.580379  606.208100  607.414572  608.081757  640.509422  641.450742  642.305345  642.985216  649.247295  650.502433  650.637281  652.894576  683.727664  684.689216  685.160812  685.533255  686.229430  686.759946  687.304426  688.088249  702.642353  703.194910  704.721208  705.711379  727.998612  729.710028  730.196413  732.363963  745.777682  746.864304  747.220220  747.655793  748.023036  749.151367  750.030594  750.818721  790.077874  791.965548  792.178364  795.076901 | 0.000000  0.178858  1.113938  1.625272  43.966536  44.358601  45.224359  45.634662  62.345382  62.439429  63.450198  63.662666  106.184871  106.478453  107.609308  107.892356  290.567861  291.627822  294.157246  296.598996  327.952719  328.138304  328.920830  329.370631  335.868742  336.883361  337.531308  338.857814  353.091137  353.592924  357.277840  357.985092  371.966206  372.283334  373.009552  373.328465  390.265970  390.454098  391.202914  391.436404  398.644193  398.968892  400.078900  400.444740  403.283539  404.061166  406.191668  408.364111  434.143129  434.416213  435.373325  435.632726  448.311939  449.138319  449.705876  450.856059  465.682248  466.145384  469.200913  469.853685  485.256420  485.456064  486.399872  488.007545  510.962436  511.225231  512.233169  512.573576  529.543133  529.731359  530.373630  531.542508  543.383571  543.845608  544.206751  545.385157  547.401419  547.854680  549.031998  549.839999  586.873153  587.605252  588.447231  589.888578  591.737341  592.239776  592.497238  593.723169  605.803679  606.221644  606.640191  607.157829  640.361276  641.396770  641.717481  642.310303  649.306585  649.677587  650.766999  652.063448  683.679121  684.333574  684.642084  685.647208  685.932612  686.319131  686.567167  687.470978  703.083653  703.371438  703.748090  704.561789  728.057114  728.811934  730.268005  731.698489  746.131600  746.396712  747.404985  747.689496  747.877882  748.478870  748.806123  749.766358  790.545565  790.819445  792.503136  793.948390 | 0.000000  0.474146  0.767563  1.296942  94.003251  94.464073  94.570986  95.069691  128.398581  129.176491  129.264827  130.054065  222.419329  222.936985  223.252490  223.854725  292.868779  294.756285  295.543752  297.429173  313.215473  313.728981  314.061081  314.741959  387.302215  388.119168  389.940838  390.830948  402.319727  404.615061  405.027997  407.239524  407.436931  407.627771  408.026800  408.422529  422.321390  423.296290  424.250093  425.175068  441.817197  442.353500  442.663551  443.178825  466.128631  466.860524  467.489201  468.384328  494.740212  495.096367  496.147786  496.511519  497.029086  497.727713  499.746042  500.544159  516.783457  517.550995  517.691969  518.611474  531.846764  532.677457  534.207044  535.044630  535.891666  536.264285  536.501669  536.910595  560.319660  560.582690  561.478936  562.055123  571.913092  572.388340  573.068084  573.489839  588.825374  589.116910  589.850935  590.276621  595.078468  595.311942  596.153617  596.701780  598.165018  599.247113  600.656594  601.890633  623.114736  623.890332  624.466972  625.355737  626.570011  627.247743  627.419112  628.162548  665.803150  666.193473  666.953405  667.458632  689.216296  689.459351  689.671483  690.473134  692.722761  693.822363  693.956581  695.005205  700.076436  701.147466  701.398667  702.570753  717.207451  717.905359  718.147099  719.132540  726.937486  727.641323  729.450056  730.269783  793.967261  795.027562  795.203532  796.544451  821.561054  822.228859  822.598931  823.458065 | 0.000000  0.848405  1.307204  2.242103  94.022211  94.650634  95.289994  96.019357  128.161151  129.083555  130.317945  131.286052  222.192462  222.828547  224.284682  225.128541  292.336047  294.281498  296.668818  298.981480  313.344278  314.166511  314.570122  315.748348  386.747598  387.665771  391.114906  392.347525  401.774573  404.119303  406.206241  407.290300  408.110556  408.563460  409.164735  409.435753  422.306396  423.954463  424.530065  426.139189  441.846606  442.655141  443.363680  444.127310  466.196385  466.928381  468.017750  469.515310  494.864050  495.745124  496.411169  496.530959  497.167582  497.471701  501.068203  502.137413  516.843240  517.724163  518.317378  519.653342  531.952633  533.235932  534.551123  535.855477  535.995954  536.459985  537.288424  537.925039  560.343227  560.620571  562.092172  563.187125  572.044971  573.026085  573.367933  574.327738  588.953284  589.695807  590.188142  591.215228  595.278277  595.454400  596.657467  597.757330  598.143459  599.951133  600.691428  603.120675  622.936384  624.318846  624.932159  626.602329  626.770609  627.506240  628.086075  629.023211  665.891738  666.745745  667.310194  668.372449  689.369716  689.540184  690.197548  691.632216  692.614014  693.961645  694.805959  696.071242  699.504871  700.632095  702.784801  704.183955  717.039427  718.013812  718.831419  720.512703  726.752428  728.524561  729.458086  731.578948  793.378301  794.529349  796.456167  798.296032  821.440454  822.500510  823.165941  824.759834 |


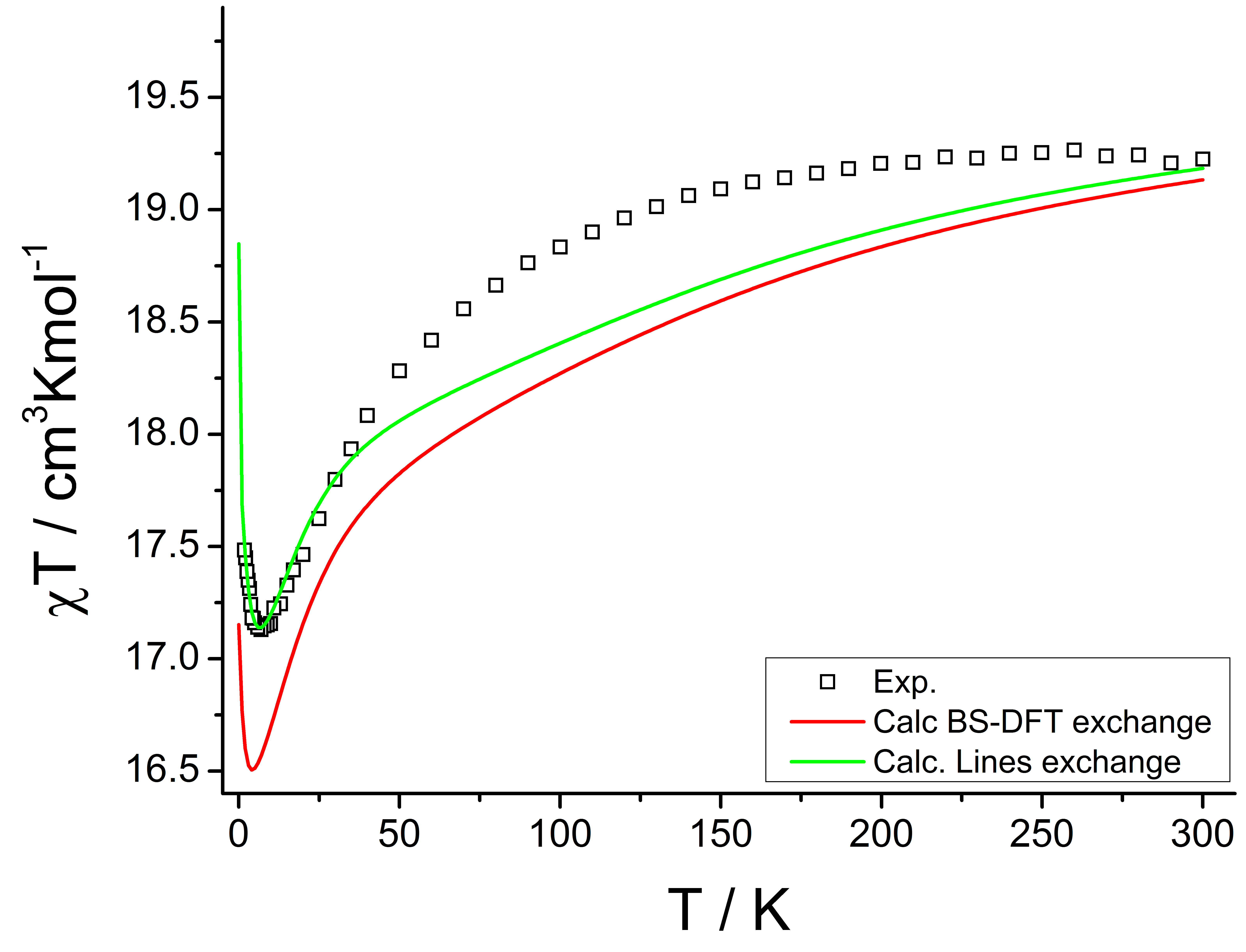

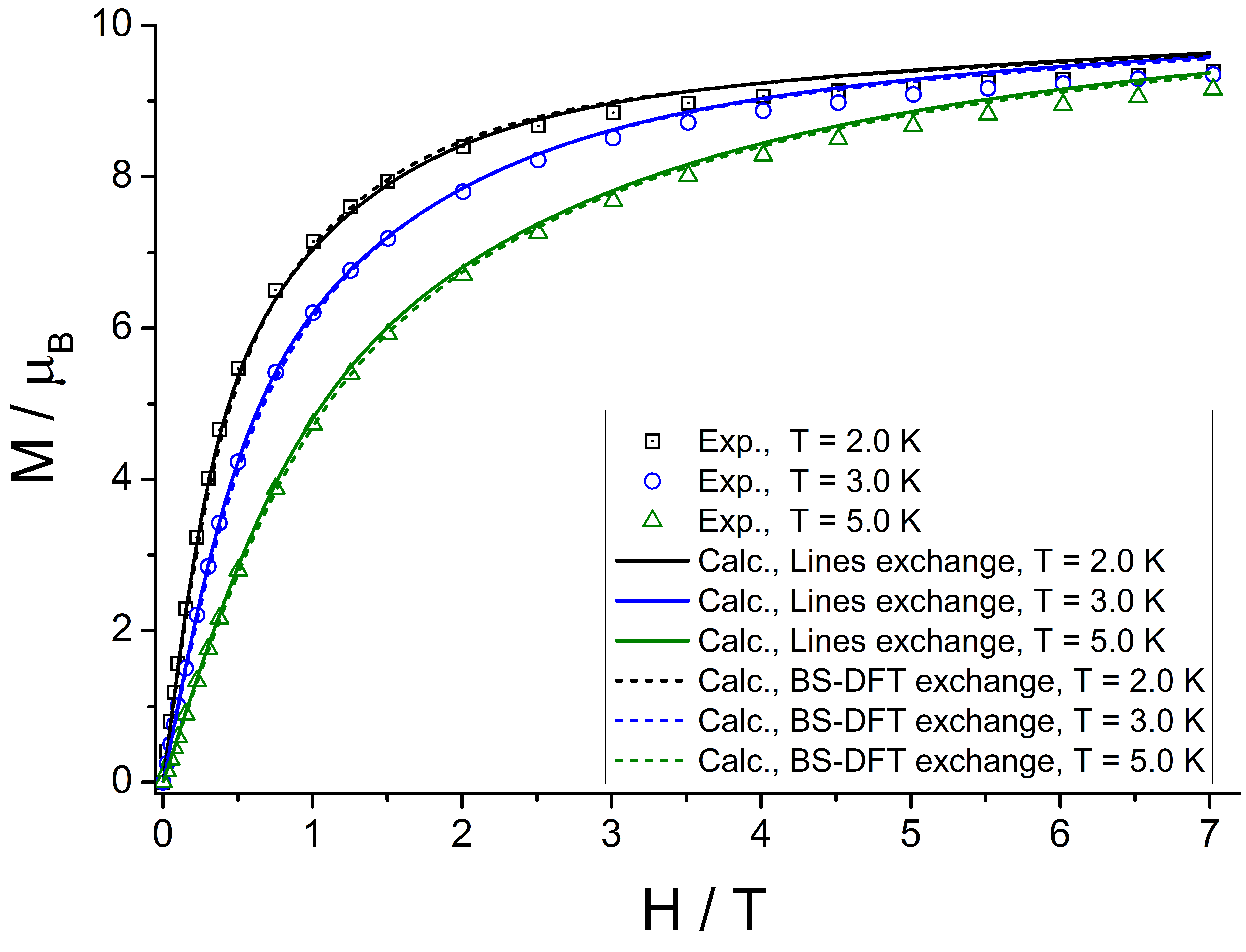


**Figure S11**: Magnetism of **1**. Only the trusted temperature interval 0-50 K was used in the fitting of the exchange parameters.


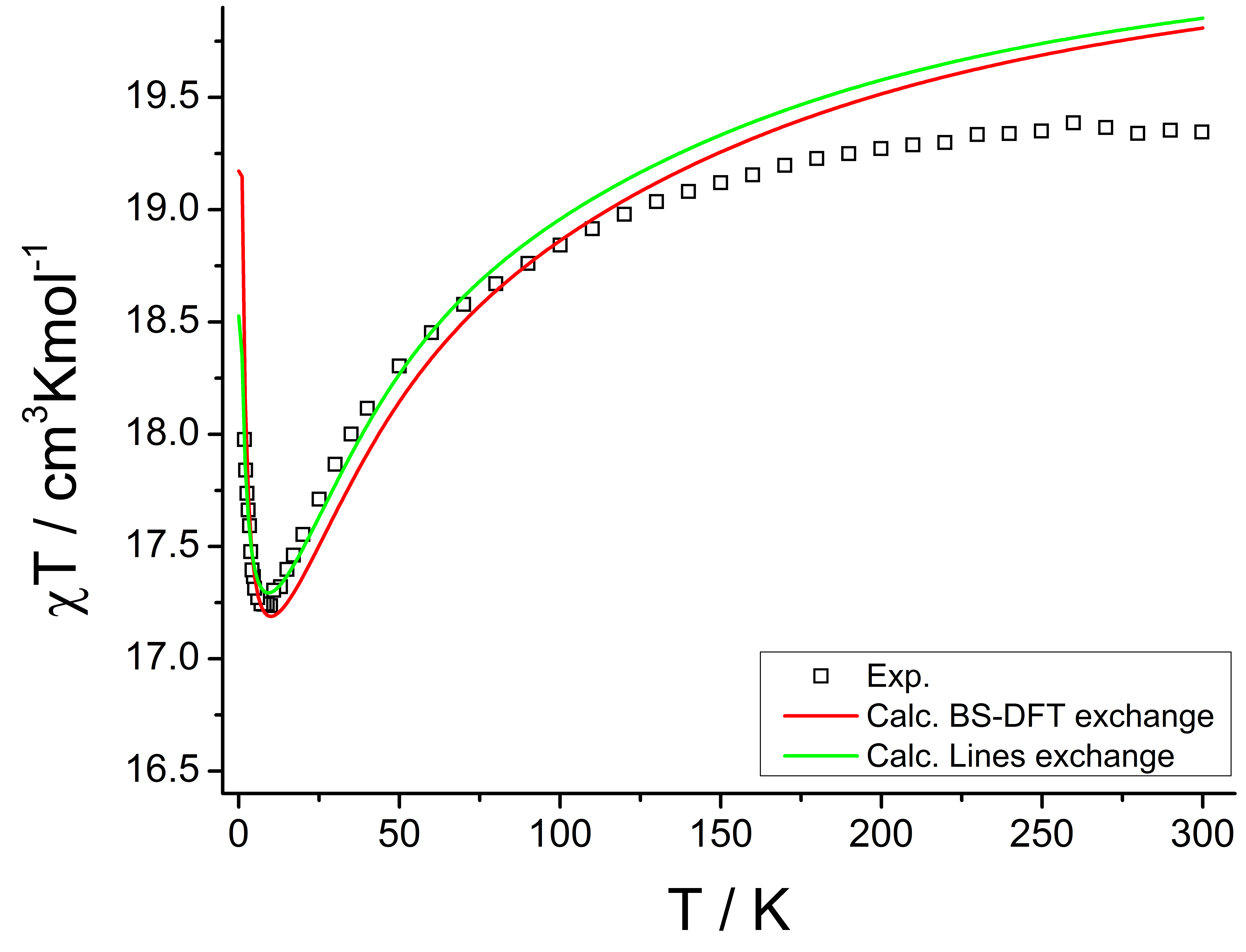

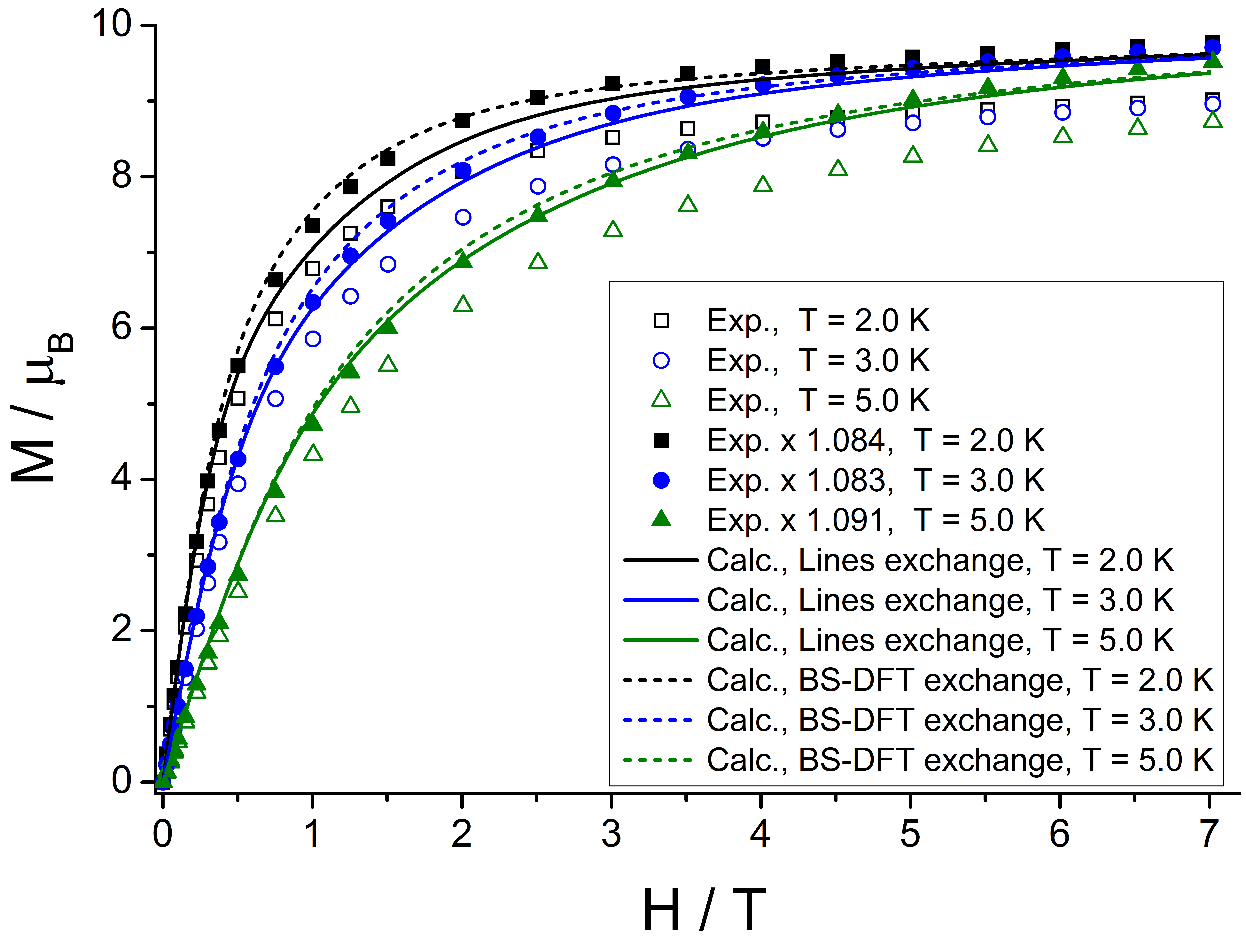


**Figure S12**: Magnetism of **1∙3H_2_O**. Only the trusted temperature interval 0-50 K was used in the fitting of the exchange parameters.

**References**

[1] ORCA-An Ab Initio, DFT and Semiempirical electronic structure package. http://www.cec.mpg.de/forschung/molekulare-theorie-und-spektroskopie/orca.html

[2] M. Shoji, K. Koizumi, Y. Kitagawa, T. Kawakami, S. Yamanaka, M. Okumura, K. Yamaguchi, *Chem. Phys. Lett.* **2006**, *432*, 343-347.

[3] V. Vieru, L. Ungur, L. F. Chibotaru, *J. Phys. Chem. Lett.* **2013**, *4*, 3565–3569.

[4] S. K. Langley, D. P. Wielechowski, V. Vieru, N. F. Chilton, B. Moubaraki, B. F. Abrahams, L. F. Chibotaru, K. S. Murray, *Angew. Chem. Int. Ed.* **2013**, *52*, 12014-12019.

[5] P.W. Anderson *Phys. Rev.*, **1959**, *115*, 2–13.

[6] M. Lines, *J. Chem. Phys.*, **1971**, *55*, 2977.
